# Study of bicyclomycin biosynthesis in *Streptomyces cinnamoneus* by genetic and biochemical approaches

**DOI:** 10.1038/s41598-019-56747-7

**Published:** 2019-12-27

**Authors:** Jerzy Witwinowski, Mireille Moutiez, Matthieu Coupet, Isabelle Correia, Pascal Belin, Antonio Ruzzini, Corinne Saulnier, Laëtitia Caraty, Emmanuel Favry, Jérôme Seguin, Sylvie Lautru, Olivier Lequin, Muriel Gondry, Jean-Luc Pernodet, Emmanuelle Darbon

**Affiliations:** 1 Institute for Integrative Biology of the Cell (I2BC), CEA, CNRS, Univ. Paris-Sud, Université Paris-Saclay, Gif-sur-Yvette, France; 2 Sorbonne Université, École Normale Supérieure, PSL University, CNRS, Laboratoire des Biomolécules, Paris, France; 3 Present Address: Unit Evolutionary Biology of the Microbial Cell, Department of Microbiology, Institut Pasteur, Paris, France; 4 Present Address: Department of Veterinary Microbiology, University of Saskatchewan, Saskatoon, Saskatchewan Canada; 5 Present Address: Frédéric Joliot Institute for Life Sciences, CEA, SPI, Saclay, France; 6 Present Address: CEA, DEN, Centre de Marcoule, Bagnols-sur-Cèze, France

**Keywords:** Microbiology, Molecular biology, Analytical biochemistry

## Abstract

The 2,5-Diketopiperazines (DKPs) constitute a large family of natural products with important biological activities. Bicyclomycin is a clinically-relevant DKP antibiotic that is the first and only member in a class known to target the bacterial transcription termination factor Rho. It derives from cyclo-(l-isoleucyl-l-leucyl) and has an unusual and highly oxidized bicyclic structure that is formed by an ether bridge between the hydroxylated terminal carbon atom of the isoleucine lateral chain and the alpha carbon of the leucine in the diketopiperazine ring. Here, we paired *in vivo* and *in vitro* studies to complete the characterization of the bicyclomycin biosynthetic gene cluster. The construction of in-frame deletion mutants in the biosynthetic gene cluster allowed for the accumulation and identification of biosynthetic intermediates. The identity of the intermediates, which were reproduced *in vitro* using purified enzymes, allowed us to characterize the pathway and corroborate previous reports. Finally, we show that the putative antibiotic transporter was dispensable for the producing strain.

## Introduction

**Figure 1:**
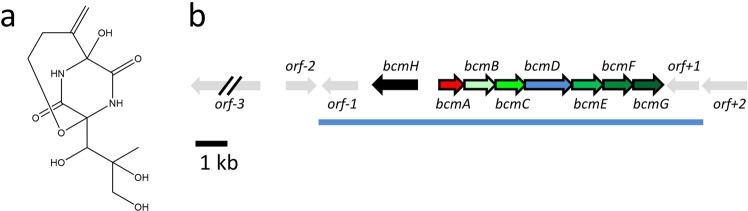
Bicyclomycin and its biosynthetic gene cluster. (**a**) Structure of the bicyclomycin molecule; (**b**) schematic representation of the bicyclomycin biosynthetic gene cluster and its flanking regions. The predicted functions of the proteins encoded by the cluster are: BcmH: MFS transporter; BcmA: cyclodipeptide synthase; BcmB, C, E, F, G: 2OG/Fe dioxygenase; BcmD: cytochrome P450 monooxygenase. The blue line indicates the extent of the insert cloned in pJWe14. The structure of the bicyclomycin molecule was drawn using ChemDraw version 18.0.0.231.

2,5-Diketopiperazines (DKPs) are a class of molecules characterized by the presence of the piperazine-2,5-dione ring obtained by the condensation of two alpha amino acids. Specialised metabolites with a DKP scaffold are produced by a wide range of microorganisms and present various interesting biological properties, including antibacterial, antifungal, antiviral or antitumoral activity^1^. Among the DKPs, bicyclomycin (**1**), also known as aizumycin or bicozamycin (Fig. 1a), is a highly oxidized, clinically-relevant molecule with a unique mechanism of action. The ability of bicyclomycin to disrupt the activity of the bacterial termination factor Rho^2,3^ has been used to treat enteropathogenic *Escherichia coli* that causes diarrhoea in humans and livestock animals^3,4^. Moreover, the use of bicyclomycin in combination with bacteriostatic concentrations of protein synthesis inhibitors dramatically enhances its bactericidal properties and potential clinical utility^5^.

Bicyclomycin was first discovered as a natural product of *Streptomyces cinnamoneus (*=*Streptomyces sapporonensis)*^6^. *Streptomyces aizunensis*^7^ and *Streptomyces griseoflavus*^8^ have since been reported as producers. In bacteria, the synthesis of the DKP core can be catalysed either by nonribosomal peptide synthetases (NRPSs) or by cyclodipeptide synthases (CDPSs)^9^. At the outset of our investigation into the biosynthesis of bicyclomycin only the first and last intermediate were proposed: cyclo-(l-isoleucyl-l-leucyl) (cIL) (**2**) and dihydrobicyclomycin (**3**), respectively^10^. During the final steps of our work, which focused on *S. cinnamoneus*, several studies on bicyclomycin biosynthesis that used *in vitro* approaches or heterologous expression were published, identifying biosynthetic genes and proposing a biosynthetic pathway for bicyclomycin in both Actinobacteria and Proteobacteria^11–13^.

Our work provides *in vivo* data that corroborate the results obtained *in vitro* on the biosynthetic pathway of bicyclomycin, establish the stereochemistry of several biosynthetic intermediates and demonstrate the dispensable nature of the transporter associated with the biosynthetic gene cluster in *S. cinnamoneus*.

## Results

### Functional analysis of the bicyclomycin biosynthetic gene cluster in *S. cinnamoneus*

Having generated and analysed a draft genome sequence of the bicyclomycin producer *S. cinnamoneus*, we had identified as candidates to direct bicyclomycin biosynthesis the same genes as those identified by other groups studying this topic^11–13^. These genes constitute a cluster composed of a CDPS gene, five genes encoding 2-oxoglutarate/iron-dependent dioxygenases (2OG/Fe dioxygenases), one gene encoding a cytochrome P450 monooxygenase, and one gene encoding a transporter belonging to the Major Facilitator Superfamily (MFS) (Fig. 1b). The cluster is flanked by genes with divergent functions that are not involved in bicyclomycin production, including genes with predicted roles in chitin degradation or the biosynthesis of an unrelated polyketide.

To provide unambiguous evidence for the involvement of the biosynthetic gene cluster (BGC) and decipher the role of individual genes in bicyclomycin biosynthesis, we attempted recombinant gene expression by introducing the complete gene cluster into *Streptomyces coelicolor* (strain M1154) or *Streptomyces lividans* (strain TK24). For these experiments, a plasmid encoding the complete *bcm* gene cluster was generated (pJWe14; Fig. 1b). The introduction of pJWe14 into M1154 or TK24 did not result in the heterologous production of bicyclomycin, even though for each strain, nine different culture media were tested (data not shown).

**Figure 2:**
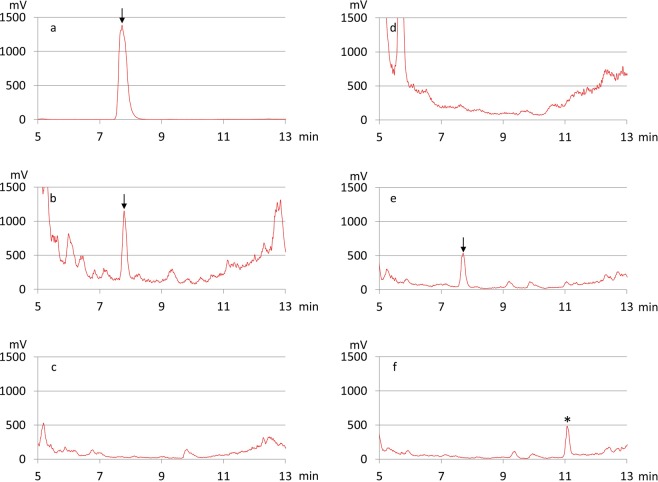
Analysis of bicyclomycin production by *S. cinnamoneus* strains. HPLC (ELSD) chromatograms obtained with method A are shown. (**a**) bicyclomycin standard; culture supernatant of *S. cinnamoneus* strains: (**b**) wild type strain; (**c**) *Δbcm::aphII* mutant strain; (**d**) *Δbcm::aphII*/pOSV668 (*Δbcm::aphII* mutant strain harbouring the empty vector); (**e**) *Δbcm::aphII*/pJWe14 (*Δbcm::aphII* mutant strain harbouring the *bcm* cluster cloned in the integrative vector pOSV668); (**f**) *Δbcm::aphII*/pMC6 (the same as (**e**), but with in-frame deletion of *bcmD*). Bacteria were grown in MP5 medium for 7 days. The bicyclomycin (**1**) peak is indicated by an arrow. The identification of bicyclomycin was confirmed by LC/MS analysis (Supplementary Table S1). * indicates a new peak in the *bcmD* deletion mutant strain.

We therefore decided to investigate the role of the *bcm* cluster directly in *S. cinnamoneus*. Accordingly, we tested ten culture media for bicyclomycin (**1**) production by S. *cinnamoneus* and observed that only growth in MP5^14^ resulted in reliable amounts detected in culture supernatants by HPLC/ELSD (Fig. 2b); the chemical standard (Santa Cruz Biotechnology, Ref. sc-391755) was used for comparison (Fig. 2a). In these culture conditions, bicyclomycin production began after two days and the amount increased over an eleven-day period (Supplementary Fig. S1). Deletion of the whole *bcm* cluster from *S. cinnamoneus* (*Δbcm::aphII* mutant, Supplementary methods) abolished bicyclomycin production (Fig. 2c). Furthermore, bicyclomycin production could be restored in the mutant *S. cinnamoneus* strain using the pJWe14 vector which carries the entire *bcm* cluster in its native configuration (Figs. 2d,e).

To study the involvement of each gene in bicyclomycin biosynthesis, we undertook their in-frame deletion. As chromosomal gene deletion is difficult and time-consuming in *S. cinnamoneus*, the pJWe14 vector appeared an excellent tool to study the *bcm* cluster. By PCR-targetting followed by excision of the resistance cassette used for gene replacement, we constructed in *E. coli* a set of pJWe14 derivatives, each of them with an in-frame deletion of one of the *bcm* biosynthetic gene, from *bcmA* to *bcmG*. To avoid as much as possible effect on downstream gene expression and to preserve translation signals and possible promoters, the first 30 nucleotides and the last 90 nucleotides of each gene were conserved in the in-frame deletion mutants. These derivatives were then introduced into the *S. cinnamoneus Δbcm::aphII* mutant. The culture supernatants of each *Δbcm::aphII* mutant harbouring modified *bcm* BGCs were analysed by HPLC/ELSD. Deletion of each of the six predicted biosynthetic genes *bcmB-G* resulted in the abolishment of bicyclomycin production. Furthermore, we constructed a vector expressing the *bcmA* gene alone under the control of its native promoter and showed that it rescued cIL (**2**) production. LC/MS analysis demonstrated that the accumulated metabolite has a retention time, m/z value and fragmentation pattern similar to an authentic standard of **2** (Supplementary Fig. S2 and Supplementary Table S1 ^15,16^). No other cyclodipeptide was detected in this experiment. Taken together, these data suggest that each of the seven genes from *bcmA* to *bcmG* is required for bicyclomycin biosynthesis. A representative chromatogram from this study is shown in Fig. 2f, chromatograms corresponding to all deletion mutants are presented in Supplementary Fig. S3.

### *In vivo* characterization of the bicyclomycin biosynthetic pathway

HPLC chromatograms corresponding to the culture supernatants of *S. cinnamoneus Δbcm::aphII* harbouring an ectopic copy of the *bcm* BGC intact or with in-frame deletion of each of the genes encoding tailoring enzymes (*bcmB*, *C*, *D*, *E*, *F*, or *G*) were analysed. They showed the appearance of additional peaks in the case of chromatograms corresponding to the different mutants (*e.g*. Fig. 2f and Supplementary Fig. S3). LC/MS/MS analyses of the same samples allowed the detection of several products whose fragmentation patterns indicate that they are related to the bicyclomycin scaffold (Supplementary Data Sheet 1). These products could be intermediates in bicyclomycin biosynthesis, as previously detected by Bradley *et al*. for intermediates **2** and **3**^10^ or shunt products resulting from the promiscuous activity of some tailoring enzymes on substrate analogues that accumulate in the absence of proper metabolic flux. Some of these products were also detected in trace amounts by LC/MS analysis in the culture supernatants of *S. cinnamoneus Δbcm::aphII* harbouring an intact copy of the *bcm* BGC. We considered that only these products were intermediates in bicyclomycin biosynthesis (Table 1).

**Table 1:** Bicyclomycin and major putative intermediates of its biosynthesis. The products detected in the culture supernatants of *S. cinnamoneus* in-frame deletion mutants are ordered by their molecular mass resemblance from cIL to dihydrobicyclomycin. -Δ indicates the loss of two H; n/a, not applicable.

Product	Molecular weight of the product [g/mol]	m/z MH+	m/z [M-H]−	Gene whose deletion is associated to the accumulation of the product	Molecular weight difference in regard to the next product and proposed modification
cIL (**2**)	226	227	—	*bcmE*	−16 g/mol (-OH)
**7**	242	243	241	*bcmC*	−16 g/mol (-OH)
**6**	258	259	257	*bcmG*	−16 g/mol (-OH)
**5**	274	275	273	*bcmB*	−14 g/mol (-OH, -Δ)
**4**/**c2**	288	289	287	*bcmD*	−16 g/mol (-OH)
dihydrobicyclomycin (**3**)	304	—	303	*bcmF*	+2 g/mol (-Δ)
bicyclomycin (**1**)	302	—	301	—	n/a

We detected **2** in the supernatants of all our constructions in varying amounts, ranging from traces to quantifiable amounts in terms of UV_214_ peak area (Supplementary Data sheet 1). Intermediate **2** specifically accumulates in the *bcmE*-deleted mutant supernatant, suggesting the involvement of BcmE in the first modification of **2**. We also detected and identified intermediate **3** thanks to its molecular weight and fragmentation pattern, consistent with previous reports (Supplementary Table S1, Supplementary Data Sheet 1)^10^. As **3** is observed specifically in the supernatant of the *bcmF*-deleted mutant, we could assign BcmF the role of dehydrogenation of the isoleucyl side chain of **3** to produce bicyclomycin (**1**). Comparison of the compounds detected in the different strain supernatants enabled us to identify two compounds of molecular weight 288 (products **4** and **c2**, Supplementary Data Sheet 1), likely to be the penultimate precursor to bicyclomycin. They are both present in trace amounts in the presence of the whole cluster and in the *bcmF*-deleted strain, and in quantifiable amounts in terms of UV_214_ peak area in the *bcmD*-deleted strain. Similarly, product **5** of molecular weight 274, is the only product found in both the whole-cluster-harbouring strain and the *bcmF*-, *bcmD*- and *bcmB*-deleted strains. It specifically accumulates in the *bcmB*-deleted strain, suggesting that BcmB catalyses the reaction from **5** to **4** (or to **c2**, see the next section). A unique compound, product **6**, whose molecular weight of 258 is compatible with the role of precursor to **5**, is found in both the whole-cluster-harbouring strain and the *bcmG*-deleted strain. Product **c4** (molecular weight of 256), which also accumulates in the *bcmG*-deleted strain was not considered *a priori* as an intermediate to bicyclomycin, as it contains a further dehydrogenation not compatible with **5** as the next intermediate. The last intermediate would be product **7** (molecular weight of 242), which accumulates in the *bcmC*-deleted strain and is also present in varying amounts in the whole-cluster-harbouring strain and the *bcmG*-, *bcmD*- and *bcmB*-deleted strains.

All together, our *in vivo* results allowed to propose the sequence in which each Bcm enzyme is involved in the pathway of biosynthesis, consistent with the generation of a highly oxidized cyclic dipeptide. This sequence is in agreement with recently reported *in vitro* studies^12^. The candidate intermediates are listed in Table 1 by ascending molecular weight values derived from LC/MS experiments (Supplementary Table S1, Supplementary Data sheet 1).

The fragmentation pattern of MS2, MS3 and MS4 data (Supplementary Data Sheet 1) also allowed making hypotheses on the reactions catalysed by Bcm enzymes, as explained in Supplementary information §1.3. However, ambiguities remained for some of these reactions (Supplementary Fig. S4). These hypotheses are compatible with data reported previously^12^.

### *In vitro* characterization of the cIL tailoring pathway and stereochemical analysis of some intermediates

**Figure 3:**
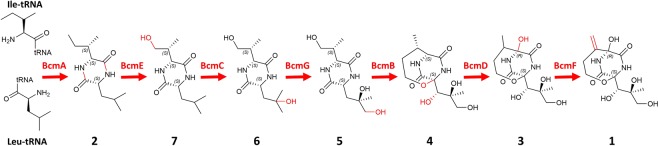
Bicyclomycin biosynthetic pathway. The modifications made at each step are indicated in red. The stereochemistry of the molecules is indicated.

In order to correlate the biosynthetic intermediates identified from *in vivo* analyses to the compounds previously identified in *in vitro* studies^12^, we characterized the activity of the tailoring enzymes BcmB, BcmC, BcmD, BcmE, BcmF and BcmG *in vitro*. An N-terminal histidine-tagged recombinant form of each protein was produced in *E. coli* and purified using nickel affinity chromatography. Single or multiple purified enzymes were reacted with cIL (**2**) or purified intermediates. The reaction mixtures and purified products were analysed by HPLC and ESI/MS, respectively (Supplementary Figs. S5–S8). The fragmentation patterns of the products obtained *in vitro* were compared to those of the products detected *in vivo* with mutant strains to check their concordance (Supplementary Table S1). The final assignments of product structures were determined by ^1^H, ^13^C NMR spectroscopy (Supplementary Information and Supplementary Figs. S9–S20). In addition to planar chemical structure elucidation, NMR analysis yielded information on the stereochemistry of compounds (Fig. 3). The observation of NOEs between Hα protons in DKP ring indicated a *syn* orientation of amino acid side chains for products **2**, **5, 6** and **7**. The determination of the configuration of new stereogenic centres in products **5** and **4** also relied on a NOE- and *J*-based NMR strategy^17–19^. The stereochemical analysis was based on the Karplus-type dihedral angle dependence of homonuclear vicinal coupling constants (^3^*J*_H,H_) and heteronuclear coupling constants (^3^*J*_C,H_). In addition, two-bond carbon-proton coupling constants (^2^*J*_C,H_) provided useful conformational and configurational information in oxygen-substituted two-carbon fragments. The results are summarized in Table 2 and detailed below. The identified compounds with their stereochemistry are positioned in the bicyclomycin biosynthetic pathway (Fig. 3).

**Table 2:** Results of *in vitro* characterization of tailoring enzymes encoded by the *bcm* cluster. “U” means unidentified products that did not correspond to any product observed in culture supernatants of the *S. cinnamoneus* strains (Supplementary data sheet 1).

Substrate	Enzyme	Observed products	Relevant Supplementary information
cIL (**2**)	BcmE	**7**	Supplementary Fig. S5B for the reaction of BcmE on **2**. Supplementary Figs. S11-S12 for NMR data for **7**.
cIL (**2**)	BcmC	U	Supplementary Fig. S5A for the reaction of BcmC on **2**.
cIL (**2**)	BcmG	U	Supplementary Fig. S5C for the reaction of BcmG on **2**.
cIL (**2**)	BcmE + BcmC	**7**, **6**	Supplementary Fig. S6A for the reaction of BcmE + BcmC on **2**. Supplementary Figs. S13-S14 for NMR data for **6**.
cIL (**2**)	BcmE + BcmG	**7**, U	Supplementary Fig. S6B for the reaction of BcmE + BcmG on **2**.
cIL (**2**)	BcmE + BcmC + BcmG	**7**, **6**, **5**, U	Supplementary Fig. S6C for the reaction of BcmE + BcmC + BcmG on **2**. Supplementary Figs. S15-S16 for NMR data for **5**. Supplementary table S6 for stereochemistry data for **5**.
cIL (**2**)	BcmE + BcmC + BcmG + BcmB	**7**, **6**, **5**, **4**, **c2**, U	Supplementary Fig. S6D for the reaction of BcmE + BcmC + BcmG+BcmB on **2**. Supplementary Figs. S17-S18 for NMR data for **4**. Supplementary table S7 for stereochemistry data for **4**.
**4**	BcmD	**3**	Supplementary Fig. S7 for the reaction of BcmD on **4**.
**3**	BcmF	bicyclomycin (**1**)	Supplementary Fig. S8 for the reaction of BcmF on **3**.

### The *in vitro* product of BcmE catalysis is **7**

We began our *in vitro* studies by focusing on the transformation of synthetic cIL by the predicted 2OG/Fe dioxygenase in the presence of iron (II) and 2-oxoglutarate. BcmE was the only 2OG/Fe dioxygenase that efficiently transformed cIL (**2**) into a novel product. MS/MS fragmentation pattern of the product of the BcmE-catalysed transformation of **2** did correspond to **7** (Supplementary Table S1), which accumulated in supernatants of the *ΔbcmC* strain. Finally, NMR analyses confirmed that BcmE catalyses the hydroxylation of cIL at the Ile Cδ to form **7**. Neither BcmF nor BcmB were able to transform cIL (data not shown). BcmC or BcmG catalysed two different and slow transformations of cIL, which was still present in the mixture after a 60 min incubation.

### **6** is the next intermediate, synthesized by BcmC

We next analysed BcmE and BcmC or BcmG reaction mixtures that contained cIL as a substrate. A new product was observed in significant quantities when BcmE and BcmC were combined and reacted with cIL (**2**). The MS/MS characteristics of this product are identical to **6**, previously observed *in vivo* when the *ΔbcmG* strain was grown in MP5. NMR analyses were used to assign **6** to the hydroxylated product of **7** at the Cγ position of Leu side chain (Fig. 3), in agreement with previous studies^12^. No compound corresponding to **c4** could be detected in *in vitro* experiments, suggesting that this compound which accumulates in large quantities in the Δ*bcmG* strain is out of the biosynthesis pathway. When BcmE was combined with BcmG, **7** was slowly converted into a new product; however, mass analysis of this product was not correlated to any metabolite observed in supernatants of *bcm* mutant strains.

### **5** is the fourth biosynthetic intermediate

As BcmG was the next enzyme in the pathway based on our *in vivo* analyses and published *in vitro* experiments^11,12^, we incubated cIL with BcmE, BcmC and BcmG, and observed that the disappearance of **6** was concomitant with the appearance of a novel product, that matched product **5** accumulating in the *bcmB*-deleted strain. The chemical structure and stereochemistry of **5** were confirmed by NMR analysis (Supplementary table S6). For **5**, the measurement of ^3^*J*_Hα-Hβ_, ^3^*J*_Hβ-CO_ and ^3^*J*_Hα-Cγ_ provided unambiguous stereospecific assignment of Hβ methylenic protons and indicated the predominance of a major Cα–Cβ rotamer of the leucyl side chain, with a χ1 angle of –60°. The analysis of ^2,3^*J*_CH_ coupling constants involving Hβ protons and Cγ, Cδ1 and Cδ2 carbons, together with NOEs is consistent with a *R* configuration of Cγ atom and a favoured rotamer around Cβ–Cγ bond adopting a χ2 angle (Cα–Cβ–Cγ–O atoms) of +60°.

### Incubation of cIL with BcmE, BcmC, BcmG and BcmB results in the bicyclic metabolite **4**

Finally, the four-enzyme mixture of BcmE, BcmC, BcmG and BcmB was able to convert **2** to **4**. The metabolite matched the one which accumulated in the *ΔbcmD* strain, and NMR analysis of the enzyme*-*generated product was used to assign the structure of **4** (Supplementary table S7). For **4**, the analysis of NOEs in combination with ^2^*J* and ^3^*J* heteronuclear coupling constants of Hβ proton with all carbon atoms of leucyl side chain are in agreement with *S* configurations of both Cα and Cβ atoms and the presence of major rotamers around Cα–Cβ and Cβ–Cγ bonds. Product **c2** was also detected in *in vitro* experiments, in much smaller quantities than **4**. It could not be isolated in quantities compatible with NMR analyses.

### **4** is efficiently transformed in dihydrobicyclomycin (**3**) by BcmD

We then incubated **4** with the cytochrome P450 BcmD in the presence of NADPH and the spinach electron transfer chain system (incubation time 24 h). BcmD catalysed the transformation of **4** to a new product with m/z value and fragmentation pattern consistent with **3** (dihydrobicyclomycin). This result confirms that **4** is a true precursor of dihydrobicyclomycin, **c2** being a by-product of the biosynthesis pathway.

### BcmF generates a vinylidene to complete the biosynthesis

Finally, when **3** was incubated with BcmF in the presence of iron (II) and 2-oxoglutarate, the molecule was transformed into a new product with m/z value and fragmentation pattern consistent with bicyclomycin (**1**), in agreement with the vinylidene formation.

### *In vivo* detection of a shunt pathway in the *bcmE* deletion mutant

We observed *in vivo* that the strain devoid of BcmE accumulates, in addition to cIL (**2**), some products in large quantities including c26, c27, c28, c29, c31 and c32 (Supplementary Data sheet 1 and Supplementary Table S2). Products c26, c27, c29, c31 and c32 have the same mass as compounds identified *in vitro* in the BcmC shunt pathway by Meng *et al*.^12^ (compounds 10, 11, 12, 14a and 14b according to their numbering). Moreover, their relative retention times in our chromatographic conditions are the same as those observed by Meng *et al*. and their fragmentation patterns are compatible with the structure proposed by Meng *et al*.^12^ Another product, c28, might be a desaturation intermediate between the products 11 and 12 characterized by Meng *et al*.^12^. These products are not detected in the other mutant strains in which BcmE is present. This indicates that, *in vivo*, in the absence of BcmE, BcmC or BcmG could act on cIL (**2**) leading to the shunt pathway observed *in vitro* by Meng *et al*.^12^.

### BcmH is dispensable for bicyclomycin export in *S. cinnamoneus*

**Figure 4:**
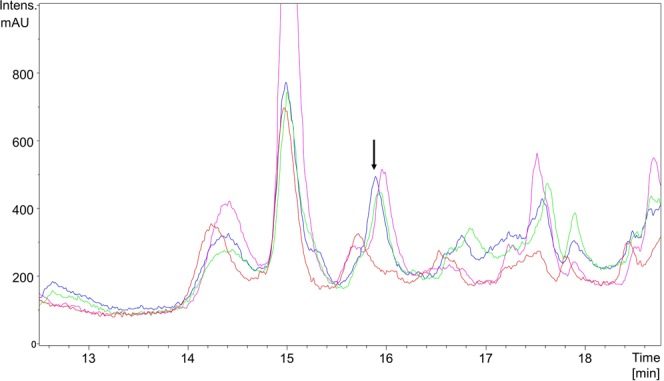
Analysis of bicyclomycin production by *S. cinnamoneus* strains. HPLC (UV, 214 nm) chromatograms obtained with method B are shown. *S. cinnamoneus Δbcm::aphII* (in red), *S. cinnamoneus Δbcm::aphII* + pMC16 (clone 1 in blue, clone 2 in green), S. *cinnamoneus Δbcm::aphII* + pMC16 + pJWe21 (in pink). The bicyclomycin (1) peak is indicated by an arrow. The fact that this peak corresponds to bicyclomycin was confirmed by LC/MS/MS analysis.

BcmH is predicted to be a MFS transporter, and when expressed in *E. coli*, BcmH confers resistance to bicyclomycin^11^. To test the role of BcmH in bicyclomycin export in the producing organism, the *bcmH* gene was inactivated on the pJWe14 plasmid, yielding pMC16. As a precautionary measure against the loss of an indispensable self-resistance gene, another plasmid, pJWe21, carrying the *bcmH* gene under the control of its native promoter was also constructed. We then attempted the construction of two strains derived from the *Δbcm* mutant: one containing only pMC16, the other containing pJWe21 and pMC16. As these two strains were obtained, we then compared bicyclomycin production by both strains. Similar amounts of bicyclomycin were found in the culture supernatants of both strains (Fig. 4). We also examined by HPLC cell-free extracts of both strains and no bicyclomycin was detected. These results indicate that BcmH is non-essential for the survival of the strain in conditions of bicyclomycin production and is also dispensable for export, which is novel in the light of previous publication^11^.

Finally, we examined the transcription of the *bcm* genes by RT-PCR analysis, using RNA extracted after seven days of cultivation, a time at which bicyclomycin is produced by *S. cinnamoneus*. The results (Supplementary Fig. S21) show that while the *bcm* biosynthetic genes (*bcmA-G*) are transcribed, *bcmH* is not. These gene expression data are in agreement with the nonessential phenotype observed for the *bcmH*-deleted strain during bicyclomycin production.

## Discussion

Our study combined genetic manipulations of the BGC for bicyclomycin that is encoded by *S. cinnamoneus* with *in vitro* enzyme characterization to complement and confirm *in vivo* the pathway proposed by Meng and coworkers^12^.

By creating and characterizing in frame gene deletions for each of the biosynthetic genes, we were able to correlate the metabolic intermediates that accumulated *in vivo* to the products of *in vitro* reactions. Our work is unique in that we deleted the full gene cluster, and replaced it with native and modified ectopic copies of it, generating robust *in vivo* data for each step in bicyclomycin biosynthesis. Moreover, we established the stereochemistry of some of the intermediates (**4** and **5**). This pathway is the most complex CDPS-dependent DKP biosynthetic pathway characterized to date with six tailoring enzymes involved in the conversion of the cyclodipeptide cIL (**2**).

Concerning the reactions catalysed by the tailoring enzymes, hydroxylations (as the one introduced by BcmC, D, E and G) are one of the canonical reactions in the repertoire of both 2OG/Fe dioxygenases and cytochrome P450 monooxygenases; desaturations (like the one catalysed by BcmF) are also well documented^20–22^. The reaction catalysed by BcmB results in hydroxylation and ether bridge formation, and is original but not unheard of. Meng *et al*.^12^ proposed that it processed through at least two BcmB-catalyzed reactions, a desaturation and an epoxidation, followed by the nucleophilic attack of the Cα carbon atom involved in the epoxyde functional group by the oxygen atom of the hydroxylated Ile Cδ.

Meng *et al*.^12^ reported that *in vitro* BcmC was more active on cIL (**2**) than BcmE, this reaction being the first of a shunt pathway involving also BcmG and BcmB. We did not observed this activity of BcmC on **2** in our *in vitro* results, as we have seen almost no substrate depletion over one hour incubation of **2** with BcmC (Supplementary Fig. S5A) and complete substrate transformation with BcmE (Supplementary Fig. S5B). The difference could be explained by different *in vitro* reaction conditions, as the buffer type, substrate and enzyme concentrations were substantially different. In particular, the substrate to enzyme ratio was 10 times lower in the conditions used by Meng *et al*.^12^. We observed *in vivo* in the *bcmE*-deleted mutant a shunt pathway similar to the one observed *in vitro* by Meng *et al*.^12^. This confirms that BcmC, BcmG and BcmB are also promiscuous *in vivo*. Patteson *et al*.^11^ also observed that overincubation of **6** with BcmG leads to the supplementary oxidation reactions performed by this enzyme that are not normally observed *in vivo*. We made similar observation *in vitro* but did not characterized the compound obtained in these conditions. Other compounds related to bicyclomycin such as **c2** or **c4**, not structurally characterized in this study, also accumulate in large quantities in some deletion mutant strains. Taken together, these observations made *in vitro* or *in vivo* indicate that some of the Bcm enzymes are promiscuous. This opens the possibility to generate novel bicyclomycin derivatives *in vivo* as well as *in vitro* by combinatorial biosynthetic approaches.

Concerning the regulation of bicyclomycin production, no putative regulatory gene is located in the vicinity of the BGC. However, bicyclomycin production is regulated, as indicated by its detection in only one out of ten growth media tested. Moreover, heterologous expression of the pathway with the native promoters (encoded by pJWe14) was not observed in *S. coelicolor* or *S. lividans*, consistent with previous attempts^12^. Only when we introduced pJWe14 into a mutant strain of *S. cinnamoneus* lacking the cluster, was bicyclomycin production observed. In contrast to attempts to express the gene cluster using the native nucleotide sequence, the addition of the strong constitutive promoter ermE*p upstream of *bcmA* did afford bicyclomycin production in *Streptomyces*^13^. All these observations suggest that a regulatory system, present in *S. cinnamoneus* and missing in the strains tested for heterologous expression, is required for the expression of the *bcm* biosynthetic genes from their native promoter(s). BGCs homologous to the *bcm* cluster are present in Actinobacteria or Proteobacteria^11,13^ and Supplementary Fig. S22. One of these homologous BGC from *P. aeruginosa* SCV20265 has been shown to direct the biosynthesis of bicyclomycin^13^. There are no putative regulators near the *bcm* cluster of *S. cinnamoneus* and no regulatory processes are known for this pathway or bicyclomycin production. More generally, a regulatory gene for CDPS-encoding BGCs has only been identified for pulcherriminic acid. This regulator is a member of the MarR family and is encoded by a gene that is adjacent to the biosynthetic genes in *Bacillus subtilis* while its homologue in *Bacillus licheniformis* is not encoded in the vicinity of the gene cluster^23–25^.

The initial assignment of *bcmH* as a resistance gene is derived from overexpression experiments performed in *E. coli*^11^. However, our results indicate that BcmH, a member of the Major Facilitator Superfamily, is not required for bicyclomycin export. Specifically, we showed that *bcmH* is dispensable in a strain producing bicyclomycin, and that it is not always transcribed at the same time as the rest of the *bcm* genes. Indeed, heterologous expression of the *bcm* cluster from *S. cinnamoneus* lacking *bcmH* in *S. coelicolor* was recently achieved^13^. An orthologue of *bcmH* is not universally conserved within or near BGCs predicted for bicyclomycin in other Actinobacteria. For instance, no *bcmH* orthologue is present near the orthologues of *bcmA-bcmG* in *Streptomyces ossamyceticus*^13^ and Supplementary Fig. S22. Other examples of transporter-encoding gene associated with specialized metabolite biosynthetic genes, but dispensable for export have already been described. For instance, in *Streptomyces venezuelae*, the *jadL* gene associated with the jadomycin BGC is dispensable for efficient jadomycin export^26^. In *Streptomyces* spp., a number of *bcmH* homologues exist, including some whose products resemble the MFS exporter isolated from a spontaneous *E. coli* mutant with resistance to bicyclomycin^27^. For example, the MFS encoded by the S*. cinnamoneus* BLA24_RS22250, which is not associated with the bcm BGC, shares 33% amino acid sequence identity with the bicyclomycin resistance protein recovered from *E. coli*. In contrast, BcmH and the *E. coli* transporter share only 20% identity. Therefore, the export of bicyclomycin in the producing organism could be due to redundant export mechanisms, one of which might involve BcmH.

It should be noted that bicyclomycin is not active against *Streptomyces* and most other actinobacteria^28,29^ (with the exception of *Micrococcus luteus*^30^) even though the ATPase activity of Rho is sensitive *in vitro* as demonstrated in the case of *S. lividans*^31^. Rho from *S. cinnamoneus* shares 78% protein sequence identity to that of *S. lividans* and all the positions predicted to be involved in the interaction with bicyclomycin^32^ are conserved between both proteins (Supplementary Fig. S23). In addition to this genus-level similarity, the *Streptomyces* proteins are identical to *E. coli* wild-type Rho at 18 of the 24 positions whose amino acid substitution confers resistance to bicyclomycin^33^. The remaining six variable positions include relatively conservative amino acid substitutions. Moreover at these six positions, the residues are identical in *S. lividans* and *S. cinnamoneus*. Based on the sequence similarities, we assume that the Rho from *S. cinnamoneus* is also sensitive to bicyclomycin. The fact that bicyclomycin production is not detrimental to the *Streptomyces* producing strain can be explained by an efficient export of the antibiotic but also by the fact that Rho might be non-essential in *Streptomyces*, as for instance in *B. subtilis*^34^.

Additional studies are required to understand the regulation of bicyclomycin biosynthesis, export, and resistance in the producing organisms. The bicyclomycin pathway is the most complex CDPS-dependent DKP biosynthetic pathway characterized so far, with six tailoring enzymes. The characterization of this pathway and the further study of its enzyme promiscuity may provide insight for the biosynthesis of bicyclomycin analogues and also for the use of these enzymes for the modification of other cyclodipeptides in combinatorial biosynthesis.

## Material and Methods

### Bacterial strains, culture conditions and strain manipulation

Bacterial strains are listed in Supplementary Table S3. *Escherichia coli* strains were manipulated using standard laboratory procedures^35,36^. When needed, the following compounds were added to *E. coli* cultures at the following concentrations: ampicillin, 100 mg/l; apramycin, 50 (liquid media) or 75 (solid media) mg/l; chloramphenicol, 30 mg/l; kanamycin, 25 mg/l; hygromycin, 50 (liquid media) or 100 (solid media) mg/l; spectinomycin, 100 mg/l; isopropyl β-D-1-thiogalactopyranoside (IPTG), 1 mM. *S. cinnamoneus* was manipulated using standard laboratory procedures^37^. Spores were prepared on Gruau medium (wheat groats, 30 g/l; glucose, 5 g/l; yeast extract, 5 g/l; pH adjusted to 7.2 with NaOH; agar, 20 g/l) (Mellouli, personal communication). Conjugations between *E. coli* ET12567pUZ8002 and *S. cinnamoneus* were carried out by the modified Kieser *et al*. protocol^37^, using MS medium complemented with 10 mM MgCl_2_ and a 3 ml antibiotic overlay of SNA medium containing fosfomycin instead of nalidixic acid. Exconjugants were patched on MS containing the selective antibiotic(s), fosfomycin and ampicillin. Antibiotic concentrations used for *Streptomyces* were as follow: ampicillin, 30 mg/l; apramycin, 30 mg/l; fosfomycin, 50 mg/l; kanamycin, 25 mg/l; hygromycin, 50 mg/l.

### Plasmids and DNA manipulations

All plasmids used in this study are listed in Supplementary Table S4. PCR reactions for cloning applications were carried out using Phusion (Thermo Fisher Scientific) or Q5 (New England Biolabs) high fidelity polymerases according to manufacturers’ protocol for GC-rich DNA, reaction mixtures being supplemented with 1 M of betaine. PCR reactions for the control of constructions and during RT-PCR were carried out using the DreamTaq DNA Polymerase (Thermo Fisher Scientific) supplemented with 1x Q Solution (Qiagen). All primers are listed in Supplementary Table S5. Primers were provided either by Integrated DNA Technologies or Eurofins. PCR products were purified using the NucleoSpin Gel and PCR Clean-up kit (Macherey-Nagel). Plasmid isolation from *E. coli* was performed using the NucleoSpin Plasmid kit (Macherey-Nagel). Restriction enzymes and DNA-modifying enzymes were supplied by Thermo Fisher Scientific. Vector DNA was systematically dephosphorylated before ligation. Digestion products were isolated on agarose gels and purified with the NucleoSpin Gel and PCR Clean-up kit (Macherey-Nagel). Ligations were performed using T4 DNA Ligase (either Thermo Fisher Scientific or Promega). Chemocompetent *E. coli* DH5α cells were used for transformation by ligation products or plasmids unless stated otherwise. *Streptomyces* genomic DNA was extracted as previously described^15^, with the exception of DNA used for genome sequencing. DNA isolation for the sequencing of the *S. cinnamoneus* ATCC 21532 genome was carried out as previously described^38^. The sequence obtained for the *bcm* gene cluster and its flanking regions were identical to those already deposited in DDBJ/ENA/GenBank^11,13^.

### Bioinformatic analyses

*In silico* analyses of sequences and primer design were made using the Clone Manager 9 Professional Edition Software (Scientific & Educational Software). Other analyses were performed using BLAST, run locally, or on the NCBI web server and AntiSMASH 2^39^. Multiple sequence alignments were done with Multalin^40^.

### Deletion of the whole *bcm* cluster

The entire bicyclomycin biosynthetic gene cluster was replaced in a double homologous recombination event with a kanamycin resistance cassette. Details of the procedure are given in Supplementary Information.

### Plasmid construction

Briefly, for the *in vivo* characterization of the bicyclomycin BGC, the *bcmA* gene with its native promoter and the whole bicyclomycin BGC were cloned into a pRT801 derivative, yielding respectively pJWe20 and pJWe14 vectors. The plasmid pJWe14 was used to generate derivatives with individual in-frame deletions for each of the *bcm* genes, yielding plasmids pMC2, pMC4, pMC6, pMC8, pMC12, pMC14 and pMC16 where the *bcmB, bcmC, bcmD, bcmE, bcmF, bcmG, bcmA* or *bcmH* gene were deleted, respectively. For the *bcmH* mutant complementation, the *bcmH* gene was cloned with its native promoter into pOSV806 vector yielding pJWe21. The plasmids obtained were transferred to appropriate *S. cinnamoneus* strains by conjugation.

For the *in vitro* characterization of decoration enzymes, genes *bcmB-G* were individually cloned into the pET15b *E. coli* expression vector. The recombinant proteins had an N-terminal 6xHis tag.

Details of cloning procedure are provided in Supplementary Information. Constructions were verified by sequencing (Genewiz).

### Chemical synthesis of cIL

Briefly, 1 g (5.5 mmoles) of H-l-Ile-OMe.HCl (Novabiochem) was dissolved in 15 ml of dimethylformamide at room temperature and 935 µl (5.5 mmoles) of diisopropylamine were added. After freezing at 4 °C, 1.37 g (5.5 mmole) of N-Boc-l-Leu-OH were added, followed by 1.13 g (5.5 mmole) of dicyclohexylcarbodiimide. After incubation for 1 h at 4 °C, the reaction mixture was incubated overnight at room temperature, and the rest of the process was performed according to^41^. 528 mg of final product (2.32 mmoles, yield 42%) was obtained, dissolved in DMSO and shown to be homogeneous by HPLC analysis using a dC18 column (Atlantis, dC18, 150 × 4.6 mm, 3 µm). The chemical structure of cIL was validated by NMR (Supplementary Information and Supplementary Figs. S9–S10).

### *In vitro* characterization of tailoring enzymes

For the *in vitro* characterization of tailoring enzymes, His-tagged proteins BcmB to G were produced in the *E. coli* BL21-AI strain and purified by nickel affinity chromatography. Enzymatic assays were performed at 30 °C in 1 ml reaction volume in the presence of 1 µM purified protein, 200 µM of diketopiperazine substrate and the necessary cofactors and co-substrates. The reaction mixtures were analysed by HPLC and ESI-MS. Reaction procedures were optimized and scaled up, and major products were purified and analysed by NMR. Details of experimental protocol are given in Supplementary Information.

### Production assays in *S. cinnamoneus* strains

All production experiments were carried out as follows. A 10 ml pre-culture in MP5 medium^14^ was launched in 50 ml Falcon tube on an orbital shaker (180 rpm) at 28 °C. The pre-culture was inoculated with either spores or mycelium fragments. After 48 h, 1 ml of pre-culture was used to inoculate 25 ml of MP5 medium in a 500 ml baffled Erlenmeyer flask, and the culture was incubated 7 days on an orbital shaker (180 rpm) at 28 °C. Supernatants were subsequently analysed by HPLC or LC-MS (for details, see Supplementary Information).

### HPLC and LC-MS analysis of the *S. cinnamoneus* culture supernatants

Culture supernatants obtained after centrifugation of the culture were filtered on 0.2 µm MiniUniPrep filter (Whatman). When following “method A”, the filtered supernatants were analyzed on an Atlantis T3 column (250 mm × 4.6 mm, 5 μm, 30 °C) using an Agilent 1200 HPLC instrument equipped with a quaternary pump. Samples were eluted with isocratic 0.1% HCOOH in H_2_O (solvent A) / 0.1% HCOOH in CH_3_CN (solvent B) (95:5) at 1 ml/min for 5 min followed by a gradient to 100% solvent B over 45 min. Molecules that were present in the supernatant were detected by an Alltech 3300 evaporative light scattering detector (ELSD) (Grace).

When necessary, an analysis of the cell content was made. The mycelium pellet obtained after centrifugation of 2 ml of culture, was washed twice with 2 ml of water, suspended in 400 µl of water and broken down with glass beads using the FastPrep-24 apparatus (MP Biomedicals). A cell-free extract was obtained after centrifugation (13,000 g, 4 °C, 30 min) to remove cell debris.

LC-MS analyses were performed using an Agilent 1100 HPLC coupled via split system to an Esquire HCT ion trap mass spectrometer (Bruker) set in positive and negative modes. Multiple analyses were made. Some of them were made using “method A”, some other using the conditions described by^15^ for CDPS 1–47 and called “method B”.

## Supplementary Information

Supplementary Information.

Supplementary Information 2.

## Data Availability

All data generated and analysed during this study are included in this published article and its supplementary information files.
