# Supplementary material for: Study of bicyclomycin biosynthesis in Streptomyces cinnamoneus by genetic and biochemical approaches
Source: Sci Rep. 2019 Dec 27;9:20226. doi: 10.1038/s41598-019-56747-7 (PMC6934819; doi:10.1038/s41598-019-56747-7)
Supplement: Supplementary file 1 — Supplementary Information. [file 41598_2019_56747_MOESM1_ESM.pdf]

Study of bicyclomycin biosynthesis in *Streptomyces cinnamoneus* by genetic and biochemical approaches

Jerzy WITWINOWSKI, Mireille MOUTIEZ[#], Matthieu COUPET[#], Isabelle CORREIA[#], Pascal BELIN[#], Antonio RUZZINI, Corinne SAULNIER, Laëtitia CARATY, Emmanuel FAVRY, Jérôme SEGUIN, Sylvie LAUTRU, Olivier LEQUIN, Muriel GONDRY, Jean-Luc PERNODET and Emmanuelle DARBON

[#] These authors contributed equally to this study.

1. Supplementary results

1.1. Kinetics of bicyclomycin production in MP5 medium (Suppl. Fig. S1)

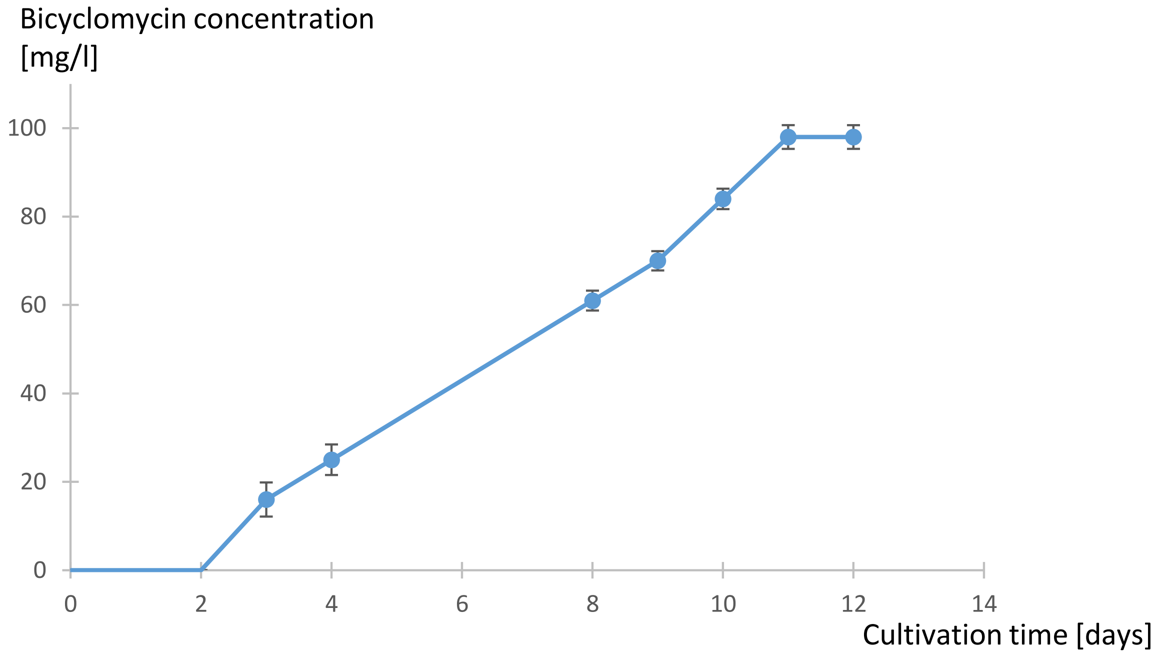

Supplementary Figure S1 Kinetics of bicyclomycin production in MP5 medium. The concentration of bicyclomycin in the culture broth was deduced from the bicyclomycin peak area in HPLC (ELSD) analysis, as compared to bicyclomycin standard of known concentration. Three cultures were analysed. The values represented are the average of the bicyclomycin concentration. Error bars represents the standard error.

1.2. Analysis of *S. cinnamoneus* mutant strains culture supernatants

1.2.1 Suppl. Fig. S2

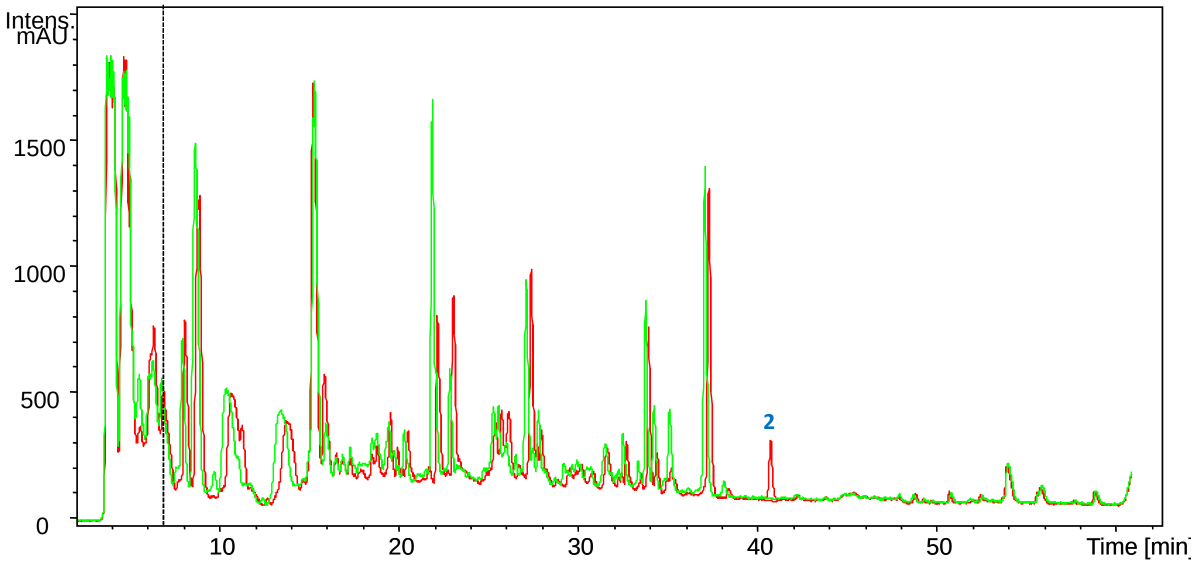

Supplementary Figure S2. HPLC (UV, 214 nm) chromatograms of *S. cinnamoneus* culture supernatants (method B). In red, culture supernatant of the Δ*bcm::aphII*/pJWe20 strain (mutant devoid of the *bcm* cluster, harbouring the *bcmA* gene under the control of its native promoter cloned into pOSV668). In green, culture supernatant of the Δ*bcm::aphII*/pOSV668 (mutant devoid of the *bcm* cluster, harbouring the empty vector). Blue number corresponds to cIL (**2**) identified by mass spectrometry. It presents an m/z of 227 in positive mode. This compound has the same retention time, m/z ratio and fragmentation pattern as a cIL (**2**) authentic standard. Culture supernatants were analysed after 7 days.

1.2.2 Suppl. Fig. S3

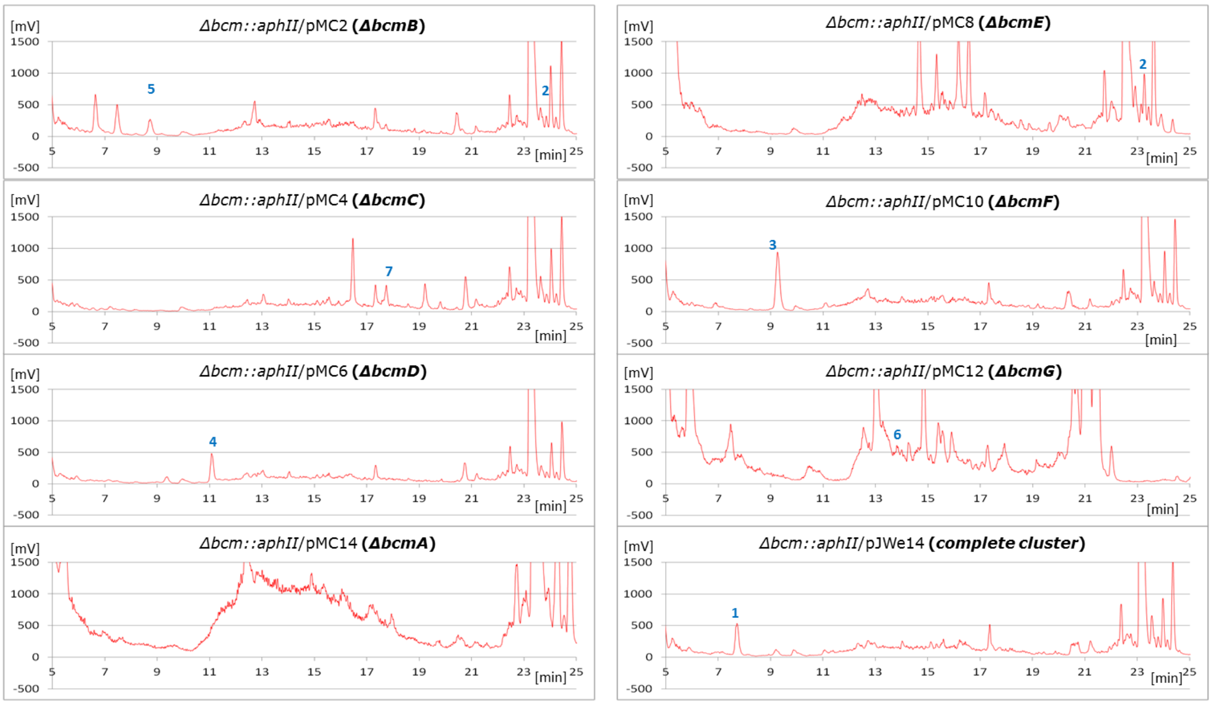

Supplementary Figure S3. HPLC (ELSD) chromatograms of *S. cinnamoneus* mutant strains culture supernatants (method A). *Δbcm::aphII* is the mutant devoid of the bicyclomycin BGC. Various integrative plasmids containing *bcm* cluster intact or bearing an in-frame deletion of only one of each gene were introduced in this mutant strain. Strain and plasmid are indicated over each chromatogram. The deleted gene is in bold. Compounds 2 to 7 correspond to the major bicyclomycin-related products accumulating in the supernatant. These compounds were also detected in trace amounts by LC/MS analysis of the supernatant of the strain containing the intact *bcm* cluster (see Supplementary data sheet 1). Culture supernatants were analysed after 7 days.

1.3. Hypothetical bicyclomycin pathway deduced from the *in vivo* analysis of intermediates

After proposing an order for the action of the tailoring enzymes, we tried to exploit the fragmentation data to make hypotheses on the precise reaction that these enzymes catalysed. Previous work [1] had shown that during the fragmentation of bicyclomycin (**1**), the first fragment to depart (m/z difference: 74) corresponds to the terminal diol of the leucyl moiety. The loss of this fragment is never observed for any of the products accumulated by the *bcmC* and *bcmG* deletion mutants. We can therefore propose that the two enzymes BcmC and BcmG add the two distal hydroxyl groups at the leucine lateral chain. However, there is no indication on the precise position modified by each of these two enzymes, hence the two possibilities proposed in Suppl. Fig. S4 (products **6a** or **6b**).

MS2, MS3 and MS4 fragmentation patterns of **7**, the product of the action of BcmE on cIL, indicates that the DKP scaffold is unmodified in this product and that the hydroxylation occurs on the side chain of either the leucyl or the isoleucyl moiety, far away enough from the DKP moiety not to modify the fragmentation pattern in all daughter species. As BcmC and G are supposed to act on the extremity of the leucine lateral chain, the most probable position of BcmE-catalysed hydroxylation is hence on the side chain extremity of the isoleucyl moiety.

Concerning the last unattributed reactions (ether bridge formation and two hydroxylations), the two enzymes BcmB and BcmD should be involved. The deletion of *bcmB* led to the accumulation of product **5** (274 g/mol, Table 1, Supplementary Data Sheet 1). The difference in molecular weight between products **4** and **5** is 14 g/mol. This could correspond to a double reaction: the closing of the ether bridge and one hydroxylation. Previous studies have shown that one hydroxylation was inhibited by a cytochrome P450 inhibitor [1]. This is consistent with our hypothesis on the involvement of the cytochrome P450 BcmD in the other hydroxylation. Our data do not allow precising the site of hydroxylation performed by BcmB and BcmD; therefore the two alternatives are presented in Supplementary Fig. S4. Taking into account the fact that BcmF catalyses the conversion of dihydrobicyclomycin (**3**) into bicyclomycin (**1**), we could propose the hypothetical biosynthetic pathway presented in Supplementary Fig. S4, with alternative hypothesis for the precise role of BcmC/BcmG and BcmB/BcmD.

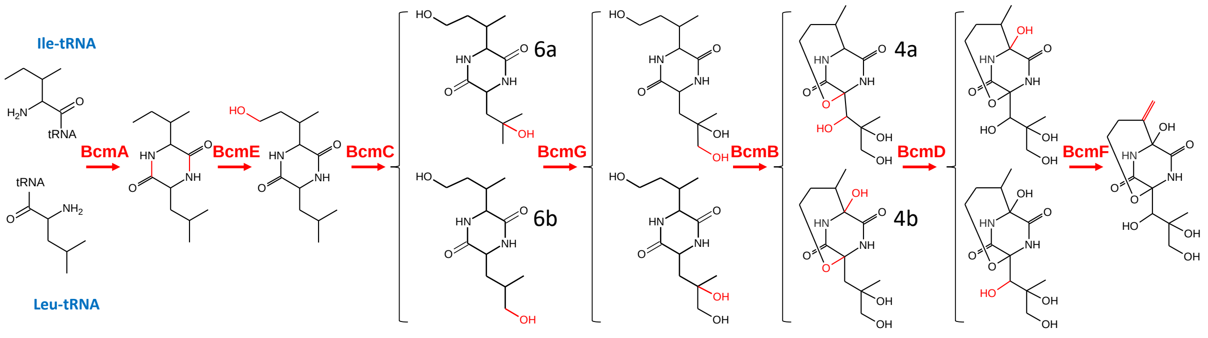

2 7 6 5 4 3 1

Supplementary Figure S4. Proposed bicyclomycin biosynthetic pathway. The modifications made at each step are indicated in red. The enzyme names are in red. The numbers below correspond to product numbers used throughout the paper. The study of intermediates produced by mutant strains did not allow choosing between the two structures for products **6** and **4**. Further *in vitro* biochemical studies confirmed this pathway and established that BcmC yielded **6a** and that BcmB yielded **4a**. The molecules were drawn using ChemDraw version 18.0.0.231.

1.4. *In vitro* characterisation of the cIL tailoring pathway

1.4.1 Suppl. Fig. S5

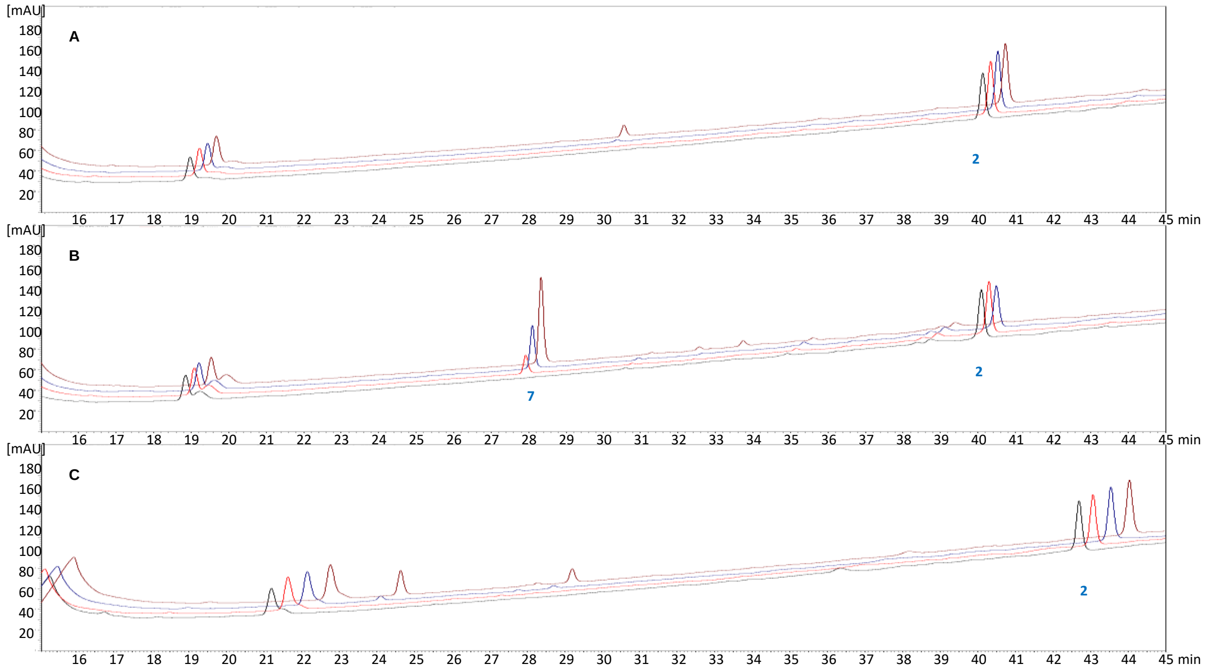

Supplementary Figure S5. HPLC (UV, 220 nm) chromatograms of *in vitro* enzymatic assays on cIL (2) (method B). (A) with BcmC, **(B)** with BcmE, **(C)** with BcmG. Chromatograms are stacked from bottom to top, the lowest corresponding to the initial state (t=0), then to reaction times of 1, 10 and 60 minutes. The position of the peaks corresponding to cIL (**2**) and product **7** is marked on the chromatogram; their identity was verified by ESI-MS.

1.4.2 Suppl. Fig. S6

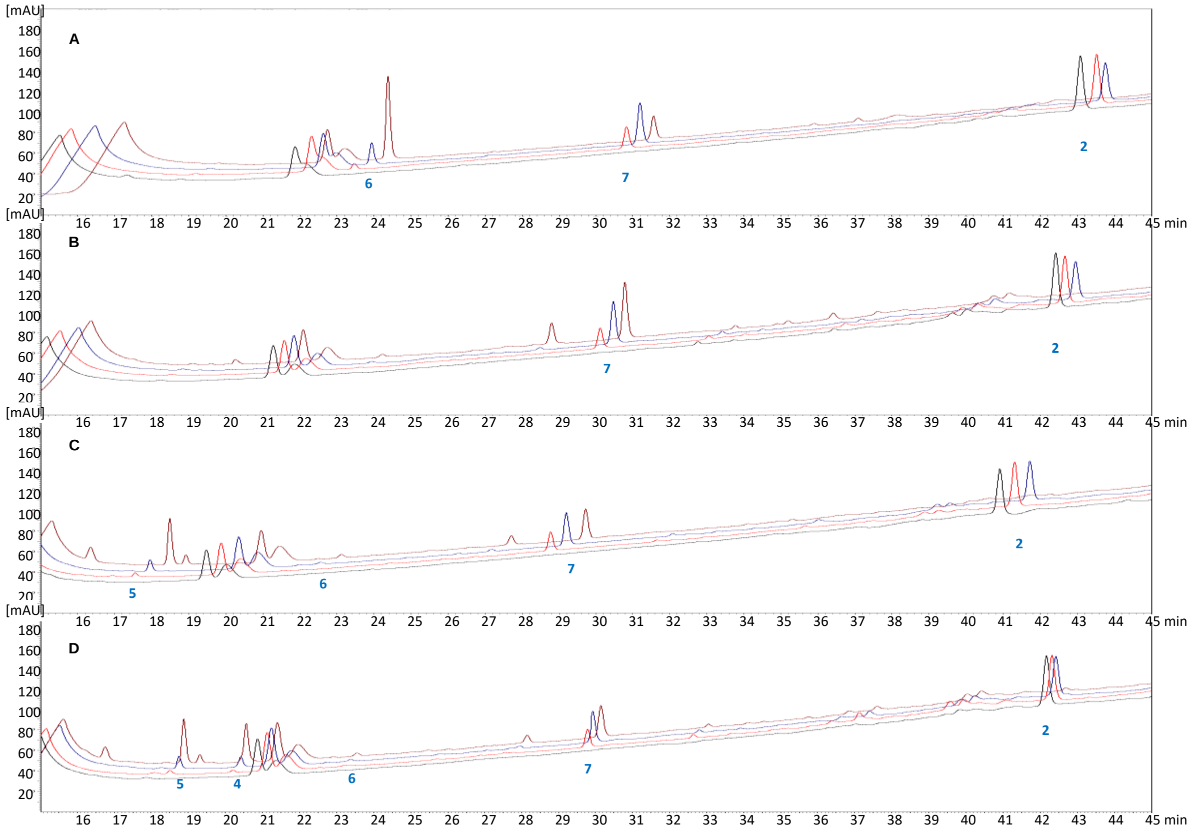

Supplementary Figure S6. HPLC (UV, 220 nm) chromatograms of *in vitro* enzymatic assays on cIL (2) (method B). (A) with BcmE+BcmC, **(B)** with BcmE+BcmG, **(C)** with BcmE+BcmC+BcmG, **(D)** with BcmE+BcmC+BcmG+BcmB. Chromatograms are stacked from bottom to top, the lowest corresponding to the initial state (t=0), then to reaction times of 3, 10 and 60 minutes. The position of the peaks corresponding to cIL (**2**) and products **4-7** is marked on the chromatogram; their identity was determined by ESI-MS.

1.4.3 Suppl. Fig. S7

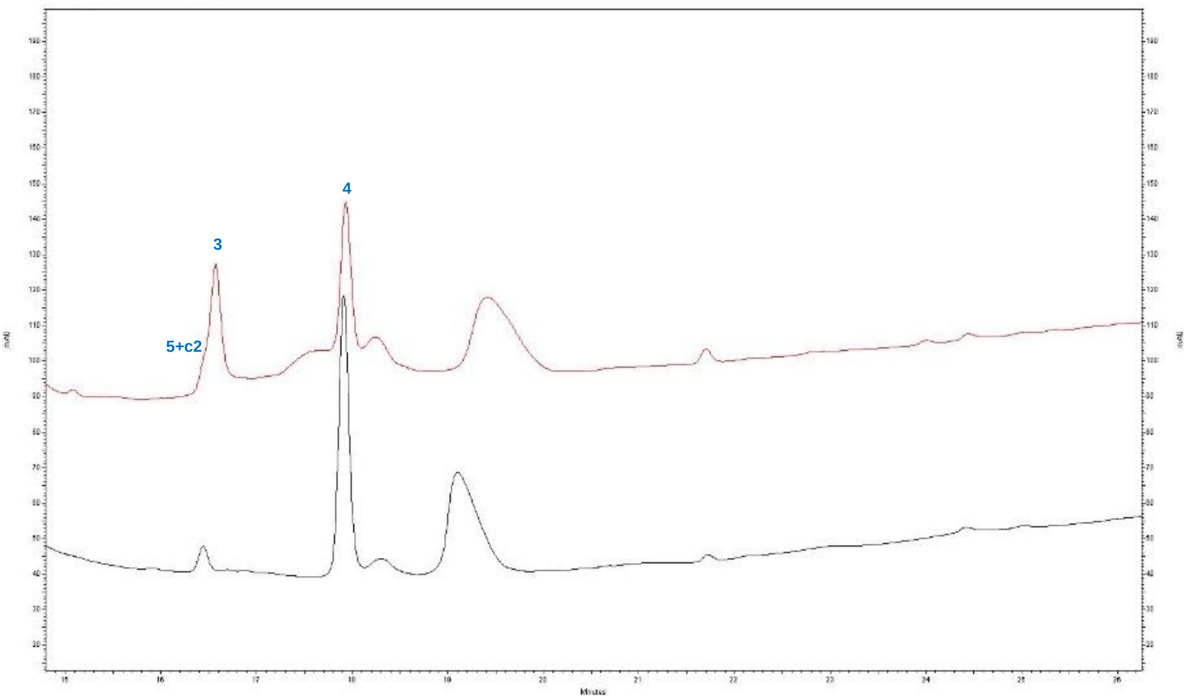

Supplementary Figure S7. HPLC (UV, 220 nm) chromatograms of *in vitro* enzymatic assay of BcmD on product 4 (method B). In black t=0, in red t=24h of incubation. Blue numbers correspond to molecule numbers used throughout the paper. Identity of molecules was determined by ESI-MS.

1.4.4 Suppl. Fig. S8

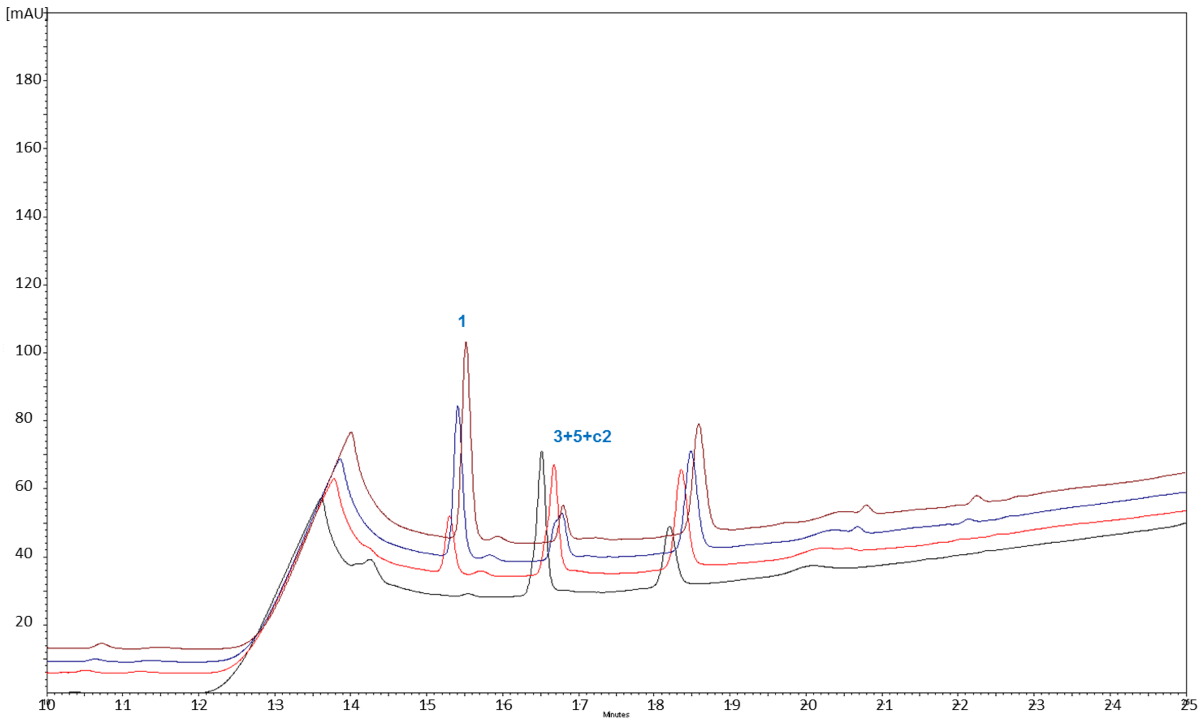

Supplementary Figure S8. HPLC (UV, 220 nm) chromatograms of *in vitro* enzymatic assay of BcmF on product 3 (dihydrobicyclomycin) (method B). Chromatograms are stacked from bottom to top, the lowest corresponding to the initial state (t=0), then to reaction times of 3, 10 and 60 minutes. Blue numbers correspond to molecule numbers used throughout the paper. Identity of molecules was determined by ESI-MS.

1.5 NMR analyses

The proposed structures of the bicyclomycin biosynthetic pathway intermediates were confirmed by NMR analysis. The products **2**, **4, 5, 6** and **7** were obtained in large quantity by the *in vitro* enzymatic reaction scale-up, purified on semi-preparative HPLC and analyzed by NMR. The ^{1}H, ^{13}C NMR assignments of the characterized compounds are given below. Products **3** and **1** were obtained in lower amounts and extracts could not be purified to homogeneity. However NMR signals of the products could be assigned using 2D correlation experiments.

1.5.1 cIL (**2**) (Suppl. Fig. S9 - S10)

cIL (**2**):

^{1}H NMR (500 MHz, DMSO): δ 8.14 (d, *J* = 2.7 Hz, 1H, H^{N} Leu), 8.01 (d, *J* = 2.5 Hz, 1H, H^{N} Ile), 3.76 (dddd, *J* = 8.6, *J* = 4.7, *J* = 2.7, *J* = 1.3 Hz, 1H, Hα Leu), 3. 69 (ddd, *J* = 4, *J* = 2.5, *J* = 1 .2 Hz, 1H, Hα Ile), 1.84 (m, 1H, Hγ Leu), 1.81 (m, 1H, Hβ Ile), 1.62 (ddd, *J* = 13.5, *J* = 8.7, *J* = 4.7 Hz, 1H, Hβ_3 Leu), 1.44 (m, 1H, Hβ_2 Leu), 1.42 (m, 1H, Hγ_{12} Ile), 1.17 (ddq, *J* = 13.4, *J* = 8.4, *J* = 7.5 Hz, 1H, Hγ_{13} Ile), 0.91 (d, *J* = 7.2 Hz, 3H, Hγ_2 Ile), 0.88 (d, *J* = 6.6 Hz, 3H, Hδ_2 Leu), 0.85 (d, *J* = 6.6 Hz, 3H, Hδ_1 Leu), 0.849 (t, *J* = 7.4 Hz, 3H, Hδ_1 Ile).

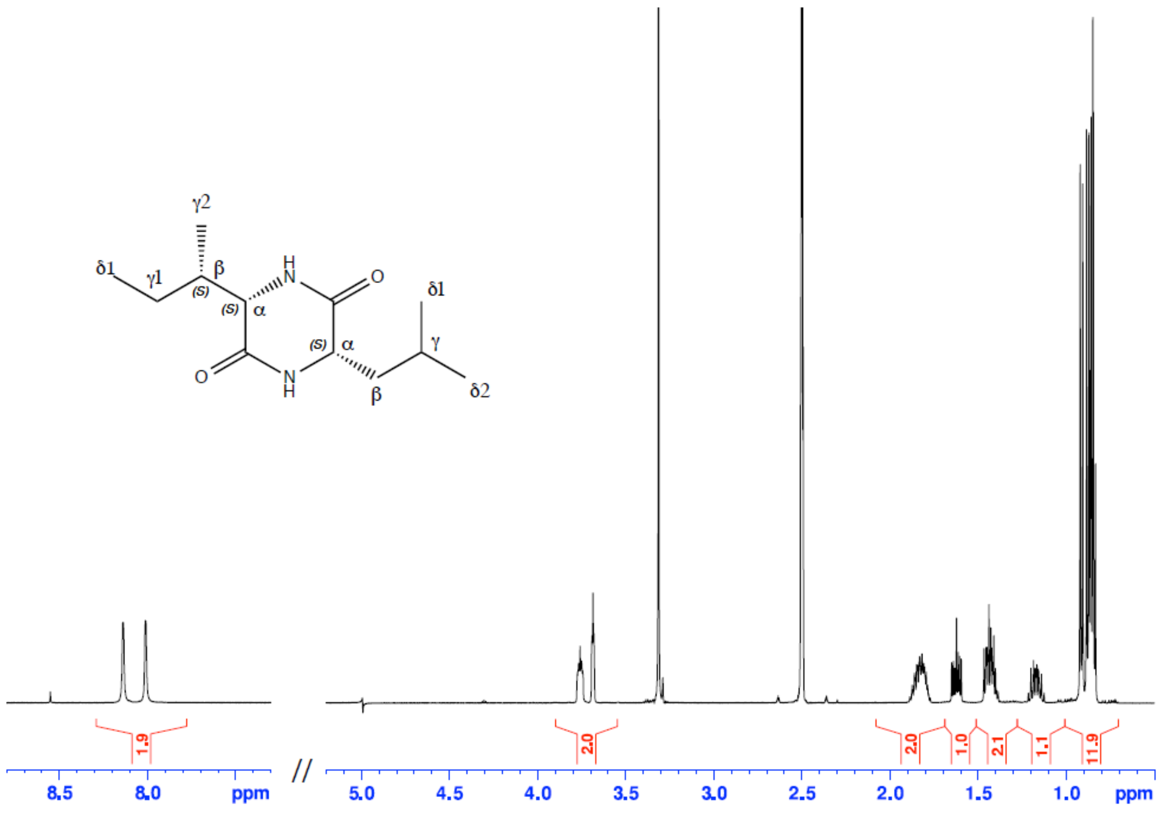

Supplementary Figure S9. 1D ^{1}H NMR spectrum of cIL (2) (500 MHz, 1.2 mg in 0.5 mL DMSO-d_6, 298.6 K)

^{13}C NMR (125 MHz, DMSO): δ 168.3 (C' Leu), 166.8 (C' Ile), 58.9 (Cα Ile), 52.3 (Cα Leu), 43.6 (Cβ Leu), 38.2 (Cβ Ile), 24.3 (Cγ_1 Ile), 23.5 (Cγ Leu), 23.1 (Cδ_2 Leu), 21.7 (Cδ_1 Leu), 15.1 (Cγ_2 Ile), 11.8 (Cγ_1 Ile).

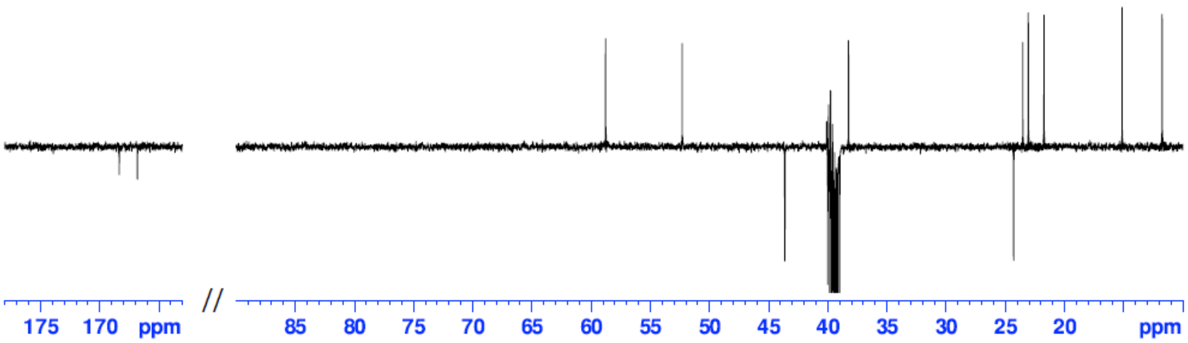

Supplementary Figure S10. 1D ^{13}C DEPTQ spectrum of cIL (2) (125 MHz, 1.2 mg in 0.5 mL DMSO-d_6, 298.6 K)

1.5.2 Product **7** (Suppl. Fig. S11 - S12)

Product **7**:

^{1}H NMR (500 MHz, DMSO): δ 8.15 (d, *J* = 2.6 Hz, 1H, H^N Leu), 8.01 (d, *J* = 2.6 Hz, 1H, H^N Ile), 4.38 (t, *J* = 5.2 Hz, 1H, HO-Cδ_1 Ile), 3.76 (dddd, *J* = 8.6, *J* = 4.7, *J* = 2.7, *J* = 1.2 Hz, 1H, Hα Leu), 3. 68 (ddd, *J* = 4, *J* = 2.7, *J* = 1 .2 Hz, 1H, Hα Ile), 3.46 (ddt, *J* = 10.5, *J* = 7.4, *J* = 5.2 Hz, 1H, Hδ_{12} Ile), 3.37 (dtd, *J* = 10.6, *J* = 7.2, *J* = 5.2 Hz, 1H, Hδ_{13} Ile), 2.04 (m, 1H, Hβ Ile), 1.85 (m, 1H, Hγ Leu), 1.63 (ddd, *J* = 13.5, *J* = 8.7, *J* = 4.7 Hz, 1H, Hβ_3 Leu), 1.56 (dtd, *J* = 13.4, *J* = 7.5, *J* = 4.4 Hz, 1H, Hγ_{13} Ile), 1.45 (ddd, *J* = 13.5, *J* = 8.6, *J* = 5.4 Hz, 1H, Hβ_2 Leu), 1.30 (dddd, *J* = 13.4, *J* = 9.6, *J* = 6.9, *J* = 5.2 Hz, 1H, Hγ_{12} Ile), 0.91 (d, *J* = 7.0 Hz, 3H, Hγ_2 Ile), 0.88 (d, *J* = 6.6 Hz, 3H, Hδ_2 Leu), 0.85 (d, *J* = 6.6 Hz, 3H, Hδ_1 Leu).

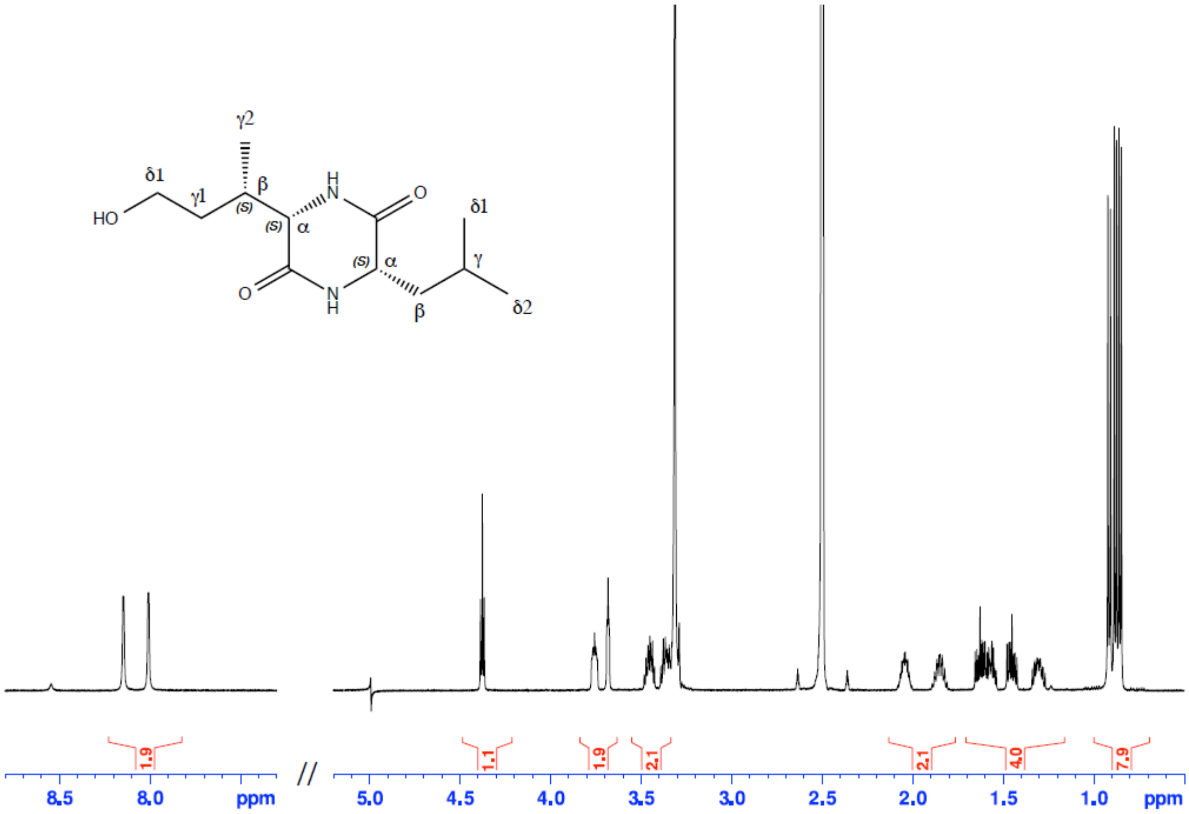

Supplementary Figure S11. 1D ^{1}H NMR spectrum of product 7 (500 MHz, 2.7 mg in 0.5 mL DMSO-d_6, 298.6 K)

^{13}C NMR (125 MHz, DMSO): δ 168.3 (C' Leu), 166.8 (C' Ile), 59.1 (Cα Ile), 58.7 (Cδ Ile), 52.3 (Cα Leu), 43.7 (Cβ Leu), 34.8 ($C\gamma_1$ Ile), 33.1 (Cβ Ile), 23.5 (Cγ Leu), 23.1 ($C\delta_2$ Leu), 21.7 ($C\delta_1$ Leu), 15.5 ($C\gamma_2$ Ile).

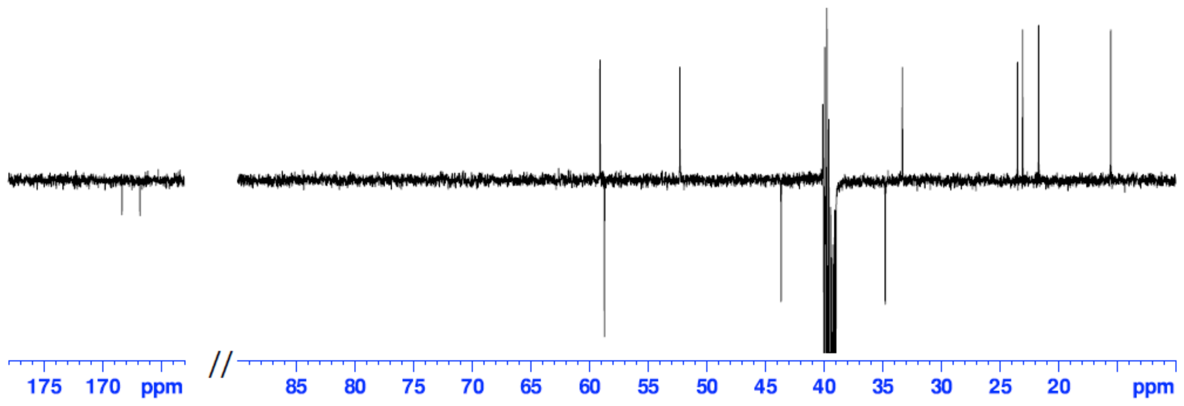

Supplementary Figure S12. 1D ^{13}C DEPTQ spectrum of product 7 (125 MHz, 2.7 mg in 0.5 mL DMSO-d_6, 298.6 K)

1.5.3 Product **6** (Suppl. Fig. S13 – S14)

Product **6:**

^{1}H NMR (500 MHz, DMSO): δ 8.07 (d, *J* = 1.8 Hz, 1H, H^N Ile), 7.50 (d, *J* = 1.5 Hz, 1H, H^N Leu), 4.95 (s, 1H, HO-Cγ Leu), 4.36 (t, *J* = 5.2 Hz, 1H, HO-$C\delta_1$ Ile), 4.10 (ddt, *J* = 9.5, *J* = 3.1, *J* = 1.5 Hz, 1H, Hα Leu), 3. 79 (dt, *J* = 3.2, *J* = 1.8 Hz, 1H, Hα Ile), 3.44 (ddt, *J* = 10.6, *J* = 7.6, *J* = 5.0 Hz, 1H, $H\delta_{12}$ Ile), 3.35 (dtd, *J* = 10.6, *J* = 7.2, *J* = 4.8 Hz, 1H, $H\delta_{13}$ Ile), 2.10 (m, 1H, Hβ Ile), 2.03 (dd, *J* = 14.2, *J* = 3.1 Hz, 1H, $H\beta_3$ Leu), 1.59 (dd, *J* = 14.3, *J* = 9.5 Hz, 1H, $H\beta_2$ Leu), 1.56 (m, 1H, $H\gamma_{12}$ Ile), 1.36 (m, 1H, $H\gamma_{13}$ Ile), 1.19 (s, 3H, $H\delta_2$ Leu), 1.15 (s, 3H, $H\delta_1$ Leu), 0.93 (d, *J* = 7.1 Hz, 3H, $H\gamma_2$ Ile).

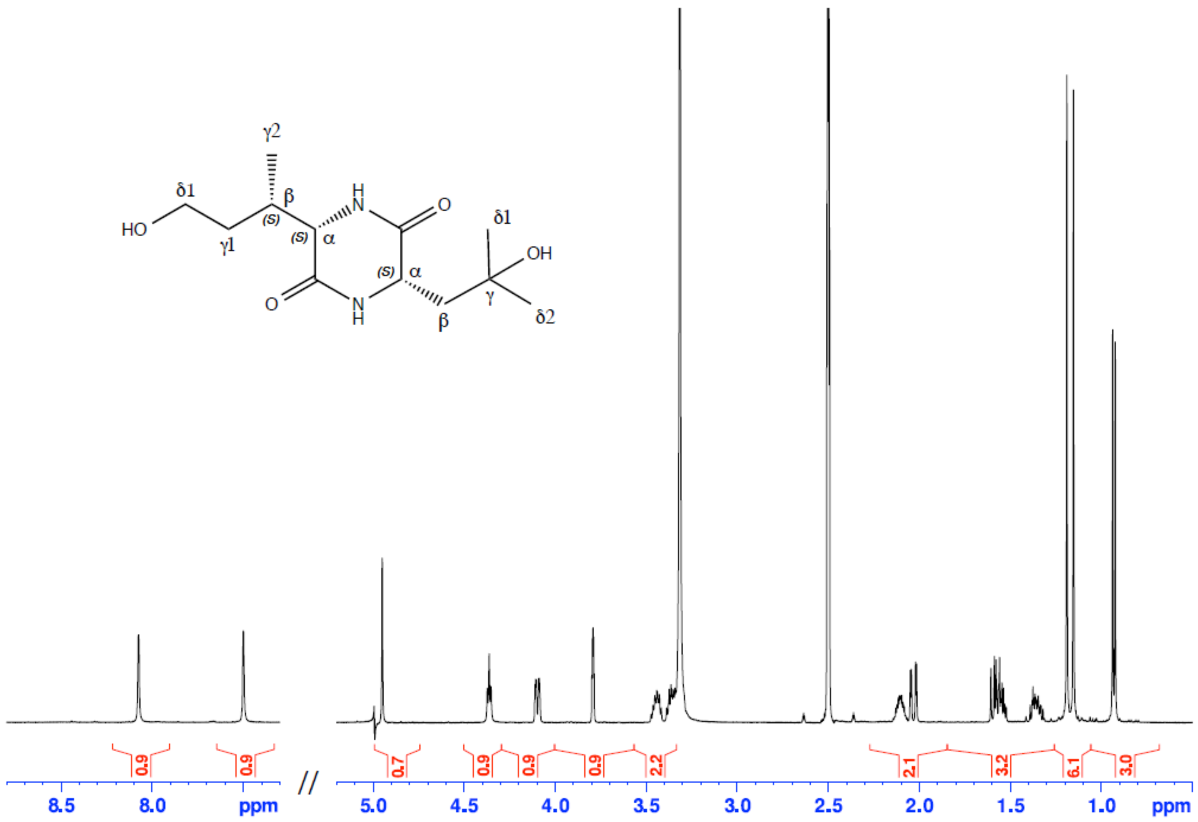

Supplementary Figure S13. 1D ^{1}H NMR spectrum of product 6 (500 MHz, 0.6 mg in 0.5 mL DMSO-d$_6$, 298.6 K)

^{13}C NMR (125 MHz, DMSO): δ 168.9 (C' Leu), 166.7 (C' Ile), 69.4 (Cγ Leu), 58.9 (Cδ_1 Ile), 58.5 (Cα Ile), 51.6 (Cα Leu), 45.0 (Cβ Leu), 34.6 (Cγ_1 Ile), 32.4 (Cβ Ile), 31.1 (Cδ_2 Leu), 27.9 (Cδ_1 Leu), 15.3 (Cγ_2 Ile).

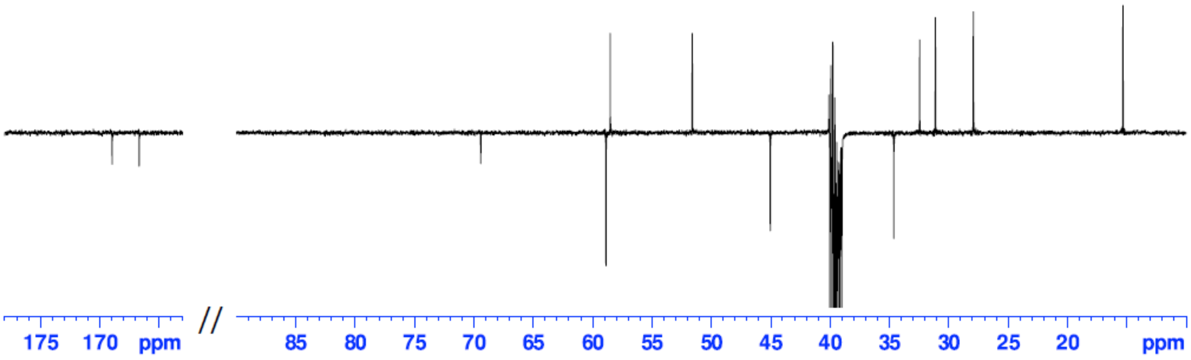

Supplementary Figure S14. 1D ^{13}C DEPTQ spectrum of product 6 (125 MHz, 0.6 mg in 0.5 mL DMSO-d_6, 298.6 K)

1.5.4 Product **5** (Suppl. Fig. S15– S16)

Product **5**:

^{1}H NMR (500 MHz, DMSO): δ 8.06 (d, *J* = 1.6 Hz, 1H, H^N Ile), 7.48 (d, *J* = 1.3 Hz, 1H, H^N Leu), 4.87 (t, *J* = 5.7 Hz, 1H, HO-Cδ_1 Leu), 4.82 (s, 1H, HO-Cγ Leu), 4.36 (t, *J* = 5.2 Hz, 1H, HO-Cδ_1 Ile), 4.12 (ddt, *J* = 9.6, *J* = 2.8, *J* = 1.4 Hz, 1H, Hα Leu), 3. 80 (dt, *J* = 2.8, *J* = 1.6 Hz, 1H, Hα Ile), 3.44 (ddt, *J* = 10.4, *J* = 7.6, *J* = 5.2 Hz, 1H, Hδ_{12} Ile), 3.35 (m, 1H, Hδ_{13} Ile), 3.25-3.17 (ABX, *J* = 10.7, J_{app} = 5.5, J_{app} = 5.8 Hz, 2H, Hδ_{12}, Hδ_{13} Leu), 2.11 (m, 1H, Hβ Ile), 2.06 (dd, *J* = 14.4, *J* = 2.7 Hz, 1H, Hβ_3 *pro-R* Leu), 1.58 (dd, *J* = 14.4, *J* = 9.6 Hz, 1H, Hβ_2 *pro-S* Leu), 1.56 (m, 1H, Hγ_{12} Ile), 1.36 (dddd, *J* = 13.4, *J* = 9.5, *J* = 7.1, *J* = 5.4 Hz, 1H, Hγ_{13} Ile), 1.08 (s, 3H, Hδ_2 Leu), 0.93 (d, *J* = 7.1 Hz, 3H, Hγ_2 Ile).

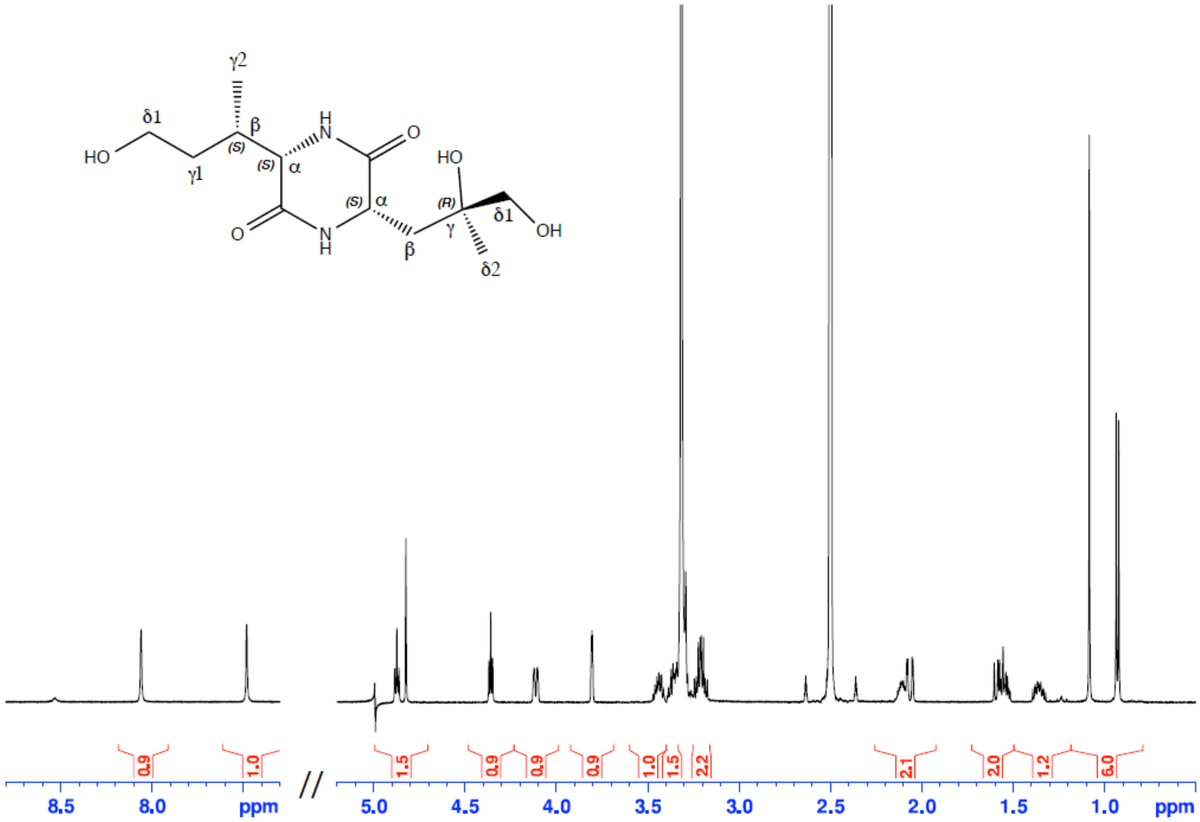

Supplementary Figure S15. 1D ^{1}H NMR spectrum of product 5 (500 MHz, 0.4 mg in 0.5 mL DMSO-d$_6$, 298.6 K)

^{13}C NMR (125 MHz, DMSO): δ 169.0 (C′ Leu), 166.6 (C′ Ile), 71.9 (Cγ Leu), 69.7 ($C\delta_1$ Leu), 58.9 ($C\delta_1$ Ile), 58.5 (Cα Ile), 51.1 (Cα Leu), 40.8 (Cβ Leu), 34.6 ($C\gamma_1$ Ile), 32.4 (Cβ Ile), 23.6 ($C\delta_2$ Leu), 15.3 ($C\gamma_2$ Ile).

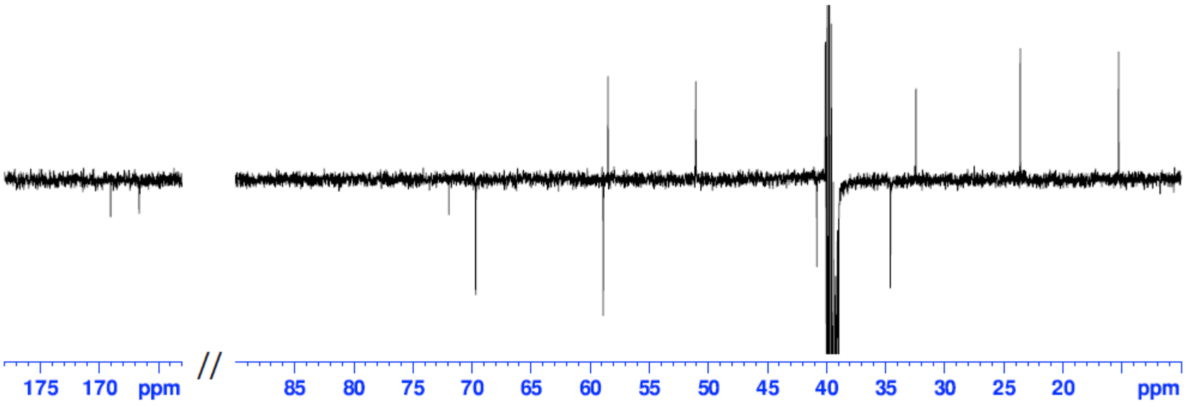

Supplementary Figure S16. 1D ^{13}C DEPTQ spectrum of product 5 (125 MHz, 0.4 mg in 0.5 mL DMSO-d_6, 298.6 K)

Stereochemical analysis of product **5** (Table S6): the large differences in $^3J_{H\alpha\text{-}H\beta}$ values (9.6 Hz and 2.8 Hz for upfield and downfield Hβ protons, respectively) indicated the predominance of a major Cα–Cβ rotamer for the leucine side chain. The measurement of $^3J_{H\beta\text{-}CO}$ and $^3J_{H\alpha\text{-}C\gamma}$ provided unambiguous stereospecific assignment of Hβ methylenic protons and determination of the χ1 angle around –60° for Cα–Cβ rotamer. This analysis was further confirmed by observed NOEs involving HN, Hα and Hβ protons (Table S6). The conformational and configurational analysis in the Cβ–Cγ fragment relied on the measurement of $^{2,3}J_{CH}$ coupling constants involving Hβ protons and Cγ, Cδ1 and Cδ2 carbons, together with NOE analysis (Table S6). The NMR data were consistent with a predominant rotamer around Cβ–Cγ bond, with a χ2 angle (Cα–Cβ–Cγ–O) around +60°, and the configuration of Cγ atom was shown to be *R*.

1.5.5 Product **4** (Suppl. Fig. S17 – S18)

Product **4**:

^{1}H NMR (500 MHz, DMSO): δ 8.73 (d, *J* = 1.5 Hz, 1H, H^N Leu), 8.40 (d, *J* = 4.2 Hz, 1H, H^N Ile), 5.11 (s, 1H, HO-Cγ Leu), 5.09 (d, *J* = 7.7 Hz, 1H, HO-Cβ Leu), 4.41 (t, *J* = 5.7 Hz, 1H, HO-Cδ_1 Leu), 3.85 (d, *J* = 7.7 Hz, 1H, Hβ Leu), 3.73 (dd, *J* = 13.5, *J* = 7.2 Hz, 1H, Hδ_{12} Ile), 3.57 (dd, *J* = 13.7, *J* = 9.4 Hz, 1H, Hδ_{13} Ile), 3.52 (dt, *J* = 4.2, *J* = 1.4 Hz, 1H, Hα Ile), 3.42 (dd, *J* = 10.9, *J* = 5.6 Hz, 1H, H$\delta_{\mathbf{12}}$ Leu), 3.29 (dd, *J* = 10.9, *J* = 5.6 Hz, 1H, H$\delta_{\mathbf{13}}$ Leu), 2.13 (m, 1H, Hβ Ile), 1.71 (ddd, 1H, *J* = 16.0, *J* = 7.2, *J* = 4.6 Hz, Hγ_{12} Ile), 1.34 (dt, *J* = 16.0, *J* = 10.0 Hz, 1H, Hγ_{13} Ile), 1.15 (s, 3H, Hδ_2 Leu), 0.95 (d, *J* = 7.0 Hz, 3H, Hγ_2 Ile).

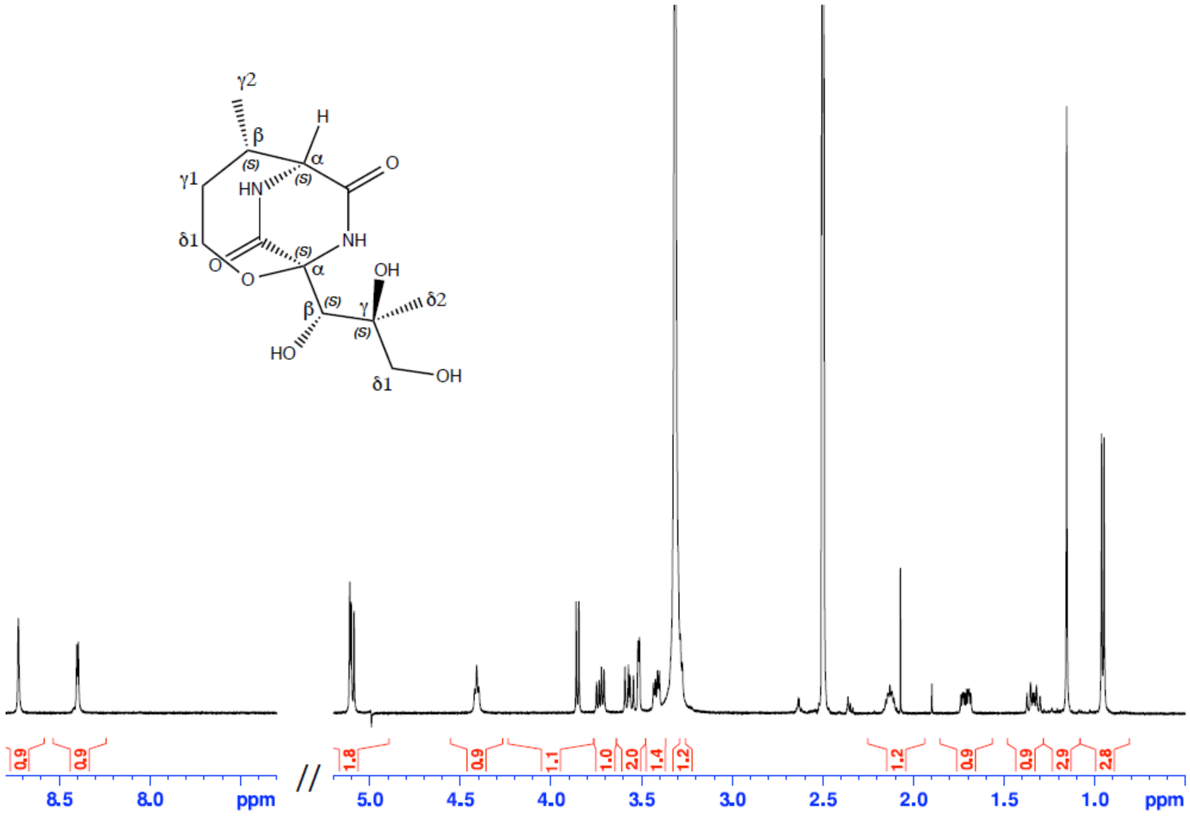

Supplementary Figure S17. 1D ^{1}H NMR spectrum of product 4 (500 MHz, < 1 mg in 0.3 mL DMSO-d$_6$, 298.6 K)

^{13}C NMR (125 MHz, DMSO): δ 171.3 (C' Ile), 168.1 (C' Leu), 86.4 (Cα Leu), 77.0 (Cγ Leu), 70.2 (Cβ Leu), 66.7 (C$δ_1$ Leu), 61.3 (C$δ_1$ Ile), 58.9 (Cα Ile), 33.5 (C$γ_1$ Ile), 40.0 (Cβ Ile), 23.9 (C$δ_2$ Leu), 19.5 (C$γ_2$ Ile).

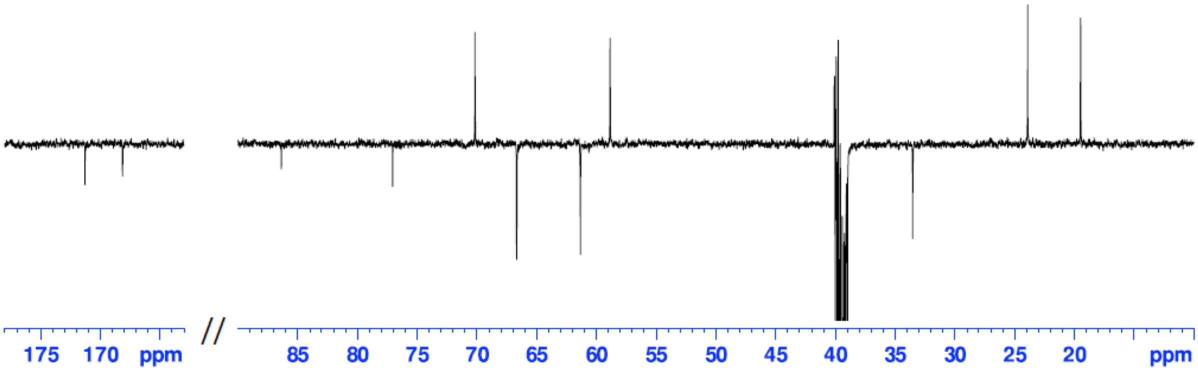

Supplementary Figure S18. 1D ^{13}C DEPTQ spectrum of product 4 (125 MHz, < 1 mg in 0.3 mL DMSO-d$_6$, 298.6 K)

Stereochemical analysis of product **4** (Table S7): the null value of $^2J_{HβCγ}$ in the Cβ–Cγ fragment of leucine side chain indicated an *anti* arrangement of Hβ proton and the hydroxyl group on Cγ carbon, while the small values of $^3J_{HβCδ1}$ and $^3J_{HβCδ2}$ allowed establishing the *gauche* arrangement of Hβ with respect to the methyl and CH_2OH substituents on Cγ. The NOE correlation observed between the methyl group on Cγ and the hydroxyl group on Cβ supported the rotamer shown in Table S7 and thus the *S* configuration for Cβ. The values of heteronuclear coupling constants involving Hβ together with the NOE correlations observed between the amide proton and the protons of the different groups on the carbon Cγ were in agreement with a major rotamer around Cα–Cβ with the *S* configuration for the Cα atom (Table S7).

1.5.6 Product **3** (Suppl. Fig. S19)

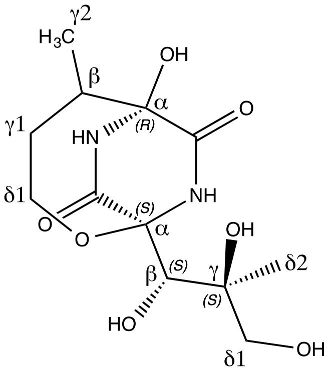

Supplementary Figure S19. Structure of product 3

^{1}H NMR (500 MHz, DMSO): δ 9.77 (s, 1H, HO-Cα Ile), 8.81 (s, 1H, H^N Leu), 8.58 (s, 1H, H^N Ile), 5.12 (d, *J* = 7.8 Hz, 1H, HO-Cβ Leu), 5.11 (s, 1H, HO-Cγ Leu), 4.46 (t, *J* = 5.8 Hz, 1H, HO-$Cδ_1$ Leu), 3.85 (d, *J* = 7.7 Hz, 1H, Hβ Leu), 3.70 (m, 1H, $Hδ_{12}$ Ile), 3.64 (m, 1H, $Hδ_{\mathbf{13}}$ Ile), 3.46 (m, 1H, $Hδ_{\mathbf{12}}$ Leu), 3.39 (m, 1H, $Hδ_{\mathbf{13}}$ Leu), 2.04 (m, 1H, Hβ Ile), 1.83 (m, 1H, $Hγ_{12}$ Ile), 1.38 (m, 1H, $Hγ_{13}$ Ile), 1.15 (s, 3H, $Hδ_2$ Leu), 0.92 (d, *J* = 7.1 Hz, 3H, $Hγ_2$ Ile).

^{13}C NMR (125 MHz, DMSO): δ 172.2 (C' Ile), 165.9 (C' Leu), 87.5 (Cα Leu), 82.1 (Cα Ile), 77.0 (Cγ Leu), 70.3 (Cβ Leu), 66.6 ($Cδ_1$ Leu), 61.3 ($Cδ_1$ Ile), 43.2 (Cβ Ile), 33.8 ($Cγ_1$ Ile), 23.9 ($Cδ_2$ Leu), 13.5 ($Cγ_2$ Ile).

1.5.7 Product **1** (Suppl. Fig. S20)

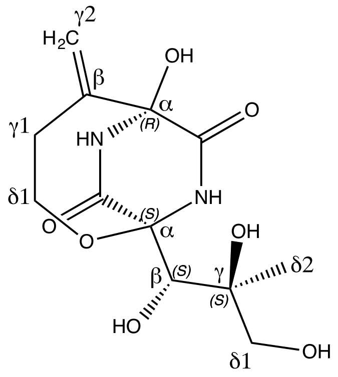

Supplementary Figure S20. Structure of product 1

^{1}H NMR (500 MHz, DMSO): δ 8.94 (s, 1H, H^N Leu), 8.67 (s, 1H, H^N Ile), 6.81 (s, 1H, HO-Cα Ile), 5.35 (d, J = 2.0 Hz, 1H, Hγ_{21} Ile), 5.24 (d, J = 7.6 Hz, 1H, HO-Cβ Leu), 5.18 (s, 1H, HO-Cγ Leu), 5.04 (m, 1H, Hγ_{22} Ile), 4.47 (t, J = 4.8 Hz, 1H, HO-Cδ_1 Leu), 3.89 (d, J = 7.6 Hz, 1H, Hβ Leu), 3.79 (ddd, J = 13.4, J = 7.1, J = 1.6 Hz, 1H, Hδ_{12} Ile), 3.61 (ddd, J = 13.0, J = 8.9, J = 1.2 Hz, 1H, Hδ_{13} Ile), 3.44 (dd, J = 11.0, J = 5.0 Hz, 1H, Hδ_{12} Leu), 3.31 (m, 1H, Hδ_{13} Leu), 2.50 (m, 1H, Hγ_{12} Ile), 2.43 (dd, J = 15.8, J = 8.9 Hz, 1H, Hγ_{13} Ile), 1.16 (s, 3H, Hδ_2 Leu).

^{13}C NMR (125 MHz, DMSO): δ 169.5 (C' Ile), 166.2 (C' Leu), 149.0 (Cβ Ile), 115.2 (Cγ_2 Ile), 87.7 (Cα Leu), 81.4 (Cα Ile), 77.1 (Cγ Leu), 70.3 (Cβ Leu), 66.6 (Cδ_1 Leu), 63.2 (Cδ_1 Ile), 35.3 (Cγ_1 Ile), 23.8 (Cδ_2 Leu).

1.6 Analysis of the transcription of the *bcm* and flanking genes (Suppl. Fig. S21)

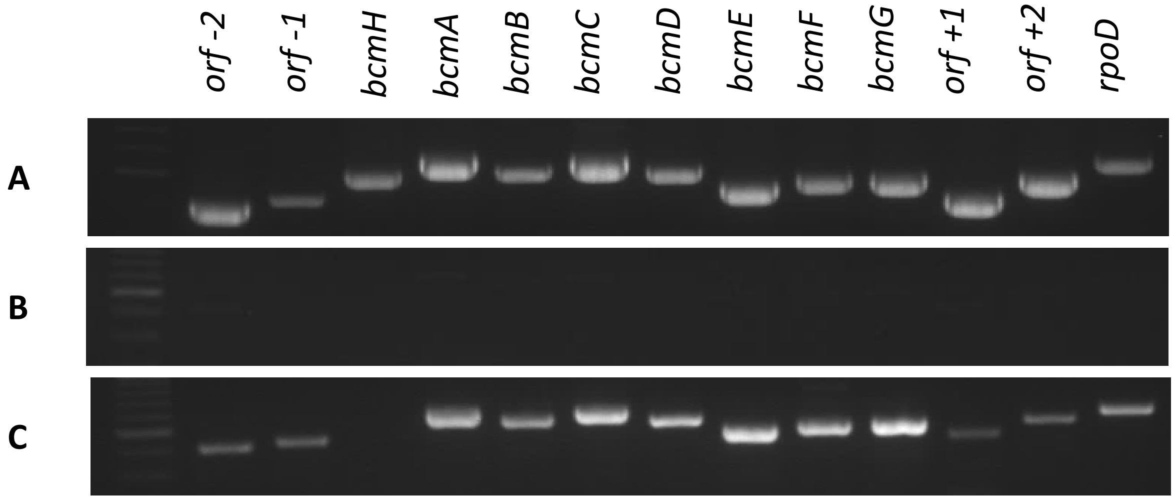

Supplementary Figure S21. Analysis of the transcription of the *bcm* and flanking genes. A: control PCR amplification on genomic DNA. B: control PCR amplification on total RNA without reverse transcription. C: RT-PCR on total RNA. DNA and RNA were extracted from the strain *S. cinnamoneus Δbcm::aphII* /pJWe14.

1.7 Homologues of the *bcm* cluster in Actinobacteria and Proteobacteria.

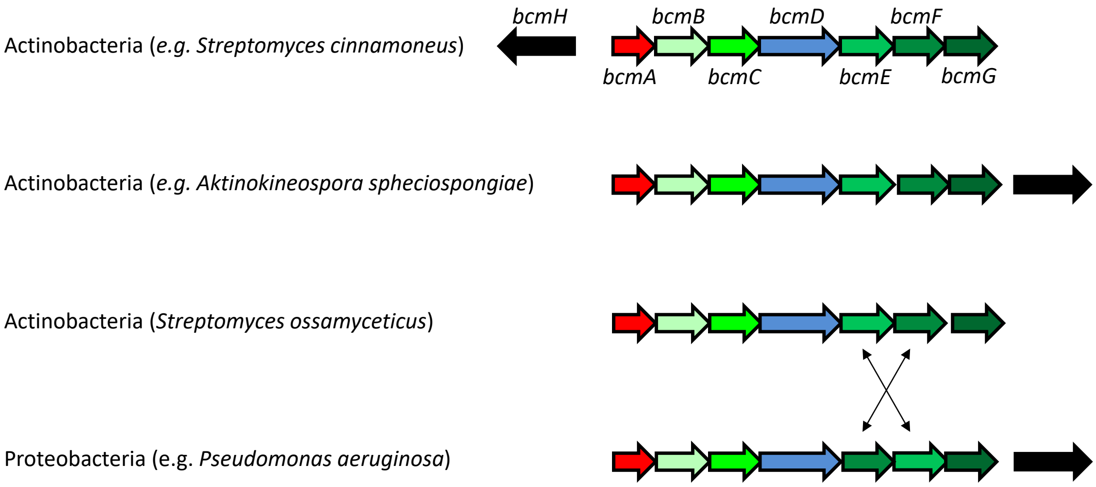

Supplementary Figure S22. Homologues of the *bcm* cluster in Actinobacteria and Proteobacteria. Identical colour fillings are used for homologous genes. Several types of gene organisation are found in Actinobacteria, with *bcmH* upstream of *bcmA*, downstream of *bcmG* or absent. In Proteobacteria, *bcmH* is found downstream of *bcmG* and the order of the homologues of *bcmE* and *bcmF* is changed.

1.8 Alignment of Rho protein sequences (Supplementary Fig. S23)

```
                1                                                     50
S.cinnamoneus   MSDTTDLMGV NAGAAGNASA PATDAPAAPA TGAATAPKRR RSGTGLDAMV
    S.lividans  MSDTTDLMG. .ARVEETAAA PATDA.SAPA TGAGS...RR RRGTGLEGMV
     M.luteus   .......... .......... .......... MTESTEQTTP TNGGGLASLK
       E.coli   .......... .......... .......... .......... ..........

                51                                                   100
S.cinnamoneus   LAELQQLASG LGIKGTARMR KGQLIEVIKE RQAGSSAAPA KADQ..APAA
    S.lividans  LAELQQVASG LGIRGTARMR KSQLIEVIKE AQA.AGGAPA KA....APAA
     M.luteus   LAQLQALASQ LGIAGGSRMR KADLVTAISD HQRGGSVADR DAAERAAQAP
       E.coli   .......... .......... .......... .......... ..........

                101                                                  150
S.cinnamoneus   SETA.ETKPK RRATSRARTG EAAAEKPAEK A......... .......AQQ
    S.lividans  ADTAGETKPK RRSTSRTRTG DEAPAEKAEK AGKADKKADK AAADKAAAQQ
     M.luteus   AAPAAETAPA AASSEDAAPA AERPARRRSR RADAD..... ......TSAP
       E.coli   .......... .......... .......... .......... ..........

                151                                                  200
S.cinnamoneus   QIEIPGQPS. ......SDEA PA....GERR RRRATA.AAG SPEPAAEAAR
    S.lividans  QIEIPGQPTP KVNASAEQAA PADDAPSERR RRRATS.DAG SPSATDTTVA
     M.luteus   AAAQDGQPQA EAREAQTEQA PRETASDQDR SGGSEARDEG EDRPQSERRS
       E.coli   .......... .......... .......... .......... ..........

                201                                                  250
S.cinnamoneus   TETRTEERTE AKAEAAADTA EGKAAKGAER QDRGERGQKG ERRERGERAE
    S.lividans  VETRAEPKAD TSAPQQSQGH Q.QGQGDARS DAEGGDGRRR DRRDRGDR.D
     M.luteus   RGRRRAGDDD AQQGQDRRSD GAQGEDGADA DRRGDREDRD DNGRENGRGR
       E.coli   .......... .......... .......... .......... ..........

                251                                                  300
S.cinnamoneus   RGDRA.ERGE RAERGQR.ER DRDRRGNRDG DAGQQQ.RGD RQQRGDRGPQ
    S.lividans  RGDRG.DRGD RGDRGDRGER GRDRRNKGDD QQNQGGGRQD RQQQGGGGRQ
     M.luteus   NGRNGRDRDN GRDRENGREN SRDRENGRDG SREQRGDKSE DGGRGDGGRG
       E.coli   .......... .......... .......... .......... ..........
```

```
               301                                              350
S.cinnamoneus  GGPQDEDDFE ...GGRRGRR GRYRDRRGRR GRDEFGANEP QLAEDDVLIP
  S.lividans   DRQQHDDGYD DDGSGRRGRR GRYRDRRGRR GRDEI..QEP QINEDDVLIP
    M.luteus   DRSRRDDRDD EGGRNRRNRR NRNERGRNRR GRGGPEVDET ELTEDDVLQP
      E.coli   MNLTELKNTP VSELITLGEN MGLENLARMR KQDIIFAILK QHAKSGEDIF

               351                                              400
S.cinnamoneus  VAGILDIL.D NYAFIRT..S GYLPGPNDVY VSLAQVRKNG LRKGDHVTGA
  S.lividans   VAGILDIL.D NYAFIRT..S GYLPGPNDVY VSLAQVRKNG LRKGDHLTGA
    M.luteus   VAGILDVL.D NYAFVRT..S GYLPGPNDVY VSLAMVKKYG LRKGDAVVGP
      E.coli   GDGVLEILQD GFGFLRSADS SYLAGPDDIY VSPSQIRRFN LRTGDTISGK

               401                                              450
S.cinnamoneus  VRQPKDGER. ........RE KFNALVRLDS VNGMAPETGR GRPEFGKLTP
  S.lividans   VRQPKEGER. ........RE KFNALVRLDS VNGMAPEHGR GRPEFNKLTP
    M.luteus   IA.PRDGEKQ QHHGGGSNRQ KFNALVKISS VNGQPAVEHP QRVEFGKLVP
      E.coli   IRPPKEGER. .......... .YFALLKVNE VNFDKPENAR NKILFENLTP

               451                                              500
S.cinnamoneus  LYPQDRLRLE TDPGV...LT TRIIDLVAPI GKGQRGLIVA PPKTGKTMIM
  S.lividans   LYPQDRLRLE TDPGV...LT TRIIDLVAPI GKGQRGLIVA PPKTGKTMIM
    M.luteus   LYPQERLRLE TDPKL...IG PRVIDLVSPI GKGQRGLIVS PPKAGKTMIL
      E.coli   LHANSRLRME RGNGSTEDLT ARVLDLASPI GRGQRGLIVA PPKAGKTMLL
                                   *              *         *      **

               501                                              550
S.cinnamoneus  QAIANAITTN NPECHLMVVL VDERPEEVTD MQRSVKGEVI SSTFDRPAED
  S.lividans   QAIANAITHN NPECHLMVVL VDERPEEVTD MQRSVKGEVI SSTFDRPAED
    M.luteus   QSIANAIKTN NPEVHLMMVL VDERPEEVTD MQRSVDGEVI ASTFDRPADD
      E.coli   QNIAQSIAYN HPDCVLMVLL IDERPEEVTE MQRLVKGEVV ASTFDEPASR
               *  ***              * **      *   *** *      *

               551                                              600
S.cinnamoneus  HTTVAELAIE RAKRLVELGH DVVVLLDSIT RLGRAYNLAA PASGRILSGG
  S.lividans   HTTVAELAIE RAKRLVELGH DVVVLLDSIT RLGRAYNLAA PASGRILSGG
    M.luteus   HTTLAELAIE RAKRLVEMGR DVVVLLDSMT RLGRAYNLAA PASGRILSGG
      E.coli   HVQVAEMVIE KAKRLVEHKK DVIILLDSIT RLARAYNTVV PASGKVLTGG
                                             *       *

               601                                              650
S.cinnamoneus  VDSTALYPPK KFFGAARNIE DGGSLTILAT ALVETGSRMD EVIFEEFKGT
  S.lividans   VDSTALYPPK RFFGAARNIE DGGSLTILAT ALVDTGSRMD EVIFEEFKGT
    M.luteus   VDSSALYPPK KFFGAARNIE NGGSLTILAT ALVETGSRMD EVIFEEFKGT
      E.coli   VDANALHRPK RFFGAARNVE EGGSLTIIAT ALIDTGSKMD EVIYEEFKGT
                           *                  *                  * *

               651                                              700
S.cinnamoneus  GNMELKLDRK LADKRIFPAV DVDPSGTRKE EILLGGEELA IVWKLRRVLH
  S.lividans   GNAELKLDRK LADKRIFPAV DVDASGTRKE EILLGSDELA ITWKLRRVLH
    M.luteus   GNMELRLSRH LAERRIFPAV DVNASGTRRE EALLSQEEVK IMWKLRRVLS
      E.coli   GNMELHLSRK IAEKRVFPAI DYNRSGTRKE ELLTTQEELQ KMWILRKIIH
                     *

               701                                   739
S.cinnamoneus  ALDSQQAIEL LLDKMKQTKS NAEFLMQIAK TTPSNGND.
  S.lividans   ALDQQQAIEL LLDKMKQTKS NAEFLIQIQK TTPTPGNGD
    M.luteus   GLEQQQALDL LTNKIKDTAS NAEFLMLVSK TTLGSKGDD
      E.coli   PMGEIDAMEF LINKLAMTKT NDDFFEMMKR S........
```

Supplementary Figure S23. Alignment of the Rho protein sequences from *S. cinnamoneus*, *S. lividans*, *M. luteus* and *E. coli*. The residues which are changed in bicyclomycin-resistant mutants of *E. coli* are marked with an asterisk. The residues which have been shown to be involved in Rho-bicyclomycin contacts with Rho from *E. coli* are highlighted in yellow.

2. Supplementary Tables

2.1. Supplementary Table S1

Compound		ionisation	m/z	fragmentation
cIL (**2**)	MW 226	MH+	**227**	69.4 (3.7); 86.2 (98.3); 98 (9.3); 114 (3.8); 153.9 (24.5); 170.8 (2.3); **181.9** (100); 198.9 (93)
cIL (2, chemically synthesized)		MH+	**227**	69.5 (1.5); 86.2 (83.9); 98.1 (4.6); 114 (3.5); 153.9 (22.1); 170.9 (2.6); **181.9** (100); 198.9 (76.9);
dihydrobicyclomycin (**3**)	MW 304	[M-H]-	**303**	99 (2.4); 154.8 (6.1); 184.9 (11.4); 185.8 (27); 186.8 (3.1); 196.8 (3.7); 210.8 (22.1); 211.9 (1.9); **228.8** (100); 258.8 (1.9); 283.9 (0.8); 284.8 (44.7)
Product **3** (*in vitro* reaction)	cIL + 5OH + Δ	[M-H]-	**303**	99.1 (2.1); 155 (3.7); 185 (6.7); 186 (11.4); 187.1 (1.3); 197 (0.7); 211.1 (10.4); 212 (0.9); **228.9** (100); 284.9 (43.1)
			303->**229**	99 (2); 182.9 (1.8); **185.8** (100); 210.8 (8.2)
bicyclomycin (**1**)	MW 302	[M-H]-	**301**	98,9 (3,1); 153,8 (3,1); 154,8 (11,2); 165,8 (2,9); 178,8 (2,9); **183,8** (100); 184,7 (12,9); 194,7 (12,3); 208,8 (21,2); 209,8 (13,2); 226,8 (39,4); 252,8 (3,1); 257,8 (5,1); 282,8 (44,2)
Product **1** (*in vitro* reaction)	cIL + 5OH + 2Δ	[M-H]-	**301**	99 (5.6); 154.9 (12.2); **183.9** (100); 184.8 (22.9); 194.8 (17); 208.9 (14.5); 209.9 (11.1); 226.9 (73.3); 257.9 (8.1); 282.9 (61.3)
			301->**184**	135.9 (26.9); **153.8** (100); 155.8 (3.1); 165.8 (67.1); 182.9 (4.1); 183.8 (4.9)
Product **7** (*in vivo* analysis of *ΔbcmC* supernatants)		MH+	**243**	196.9 (11.4); **224.9** (100)
			243->**225**	86.1 (3.6); 111.9 (9.1); 140.8 (3.4); 178.9 (2.6); 179.9 (5.9); **196.9** (100)
		[M-H]-	**241**	127.9 (7.2); 138.9 (5.9); 155.8 (3.4); 167.9 (10); 168.9 (8.4); 180.9 (32.6); **197.8** (100); 210.8 (9.6); 222.8 (30.5)
Product **7** (*in vitro* reaction)	cIL + OH	MH+	**243.1**	197.1 (10.7); **225.1** (100)
	MW 242		243->**225**	112.1 (7.4); 180 (2.6); **197.1** (100)
		[M-H]-	**241**	128 (9.1); 138.9 (4); 155.9 (3.8); 167.9 (6.7); 168.9 (6.5); 180.9 (30); 197.9 (100); 210.9 (10.1); 222.9 (28.3)
Product **6** (*in vivo* analysis of *ΔbcmG* supernatants)		MH+	**259**	129.9 (2.4); 184.9 (1.9); 224 (2.2); **240.9** (100)

			259->**241**	84.2 (84.9); 85.2 (21.6); 95.1 (21.3); 102 (11.4); 111 (21); 112 (69.5); 128.9 (23.2); 129.9 (8); 138.9 (41.5); 151.9 (8.3); 156.9 (32.6); 166.9 (83.6); 184.9 (45.8); 194.9 (11.1); **222.9** (100); 223.9 (95.4)
		[M-H]-	**257**	98 (37.3); 126 (11.5); 127.9 (6.3); 138 (3.3); 138.9 (37.9); 144.9 (15.5); **155.9** (100); 180.9 (21.9); 183.9 (95.5); 195.9 (5.6); 198.8 (78); 213.9 (77.8); 238.9 (16.1)
Product **6** (*in vitro* reaction)	cIL + 2OH MW 258	MH+	**259.1**	130 (5); 185 (2.1); 224 (1); **241.1** (100)
			259->**241**	84.4 (51.7); 85.3 (6.9); 95.2 (11.2); 102.2 (7.4); 111.2 (9.9); 112.1 (37); 129.1 (20.8); 130.1 (6.1); 139.1 (30.7); 157 (31.6); 167 (52.4); 185 (47.3); 195 (8.2); **223.1** (100); 224 (89.1)
		[M-H]-	**257**	98.1 (40.9); 126 (14.8); 138 (7); 138.9 (34); 144.9 (17.7); **155.9** (100); 180.9 (17.6); 183.9 (87.2); 196 (7); 198.9 (82.7); 213.9 (71.7); 238.9 (19.1)
Product **5** (*in vivo* analysis of *ΔbcmB* supernatants)		MH+	**275**	82.1 (3.8); 85.1 (4.7); 102 (8.6); 126.9 (22.1); 129.9 (8.7); 145.8 (2.6); 210.9 (2.4); 238.9 (23.6); **256.9** (100)
			275->**257**	82.2 (26.5); 84.2 (3.8); 85.2 (12.7); 102 (5.3); **126.9** (100); 129.8 (9.5); 193.9 (5.1); 210.9 (4.3); 220.9 (7); 238.9 (63.6); 239.8 (3.3)
		[M-H]-	273	82.2 (9.9); 98 (27.2); 125.9 (9.9); 138.9 (25.7); 143.9 (18.2); 144.9 (27.7); 155.8 (97.8); 180.8 (15.7); **198.8** (100); 199.8 (13.3); 210.8 (20); 224.9 (13); 229.9 (25.5); 236.8 (50.3); 254.8 (15.6)
Product **5** (*in vitro* reaction)	cIL + 3OH MW 274	MH+	**275.1**	82.3 (1.8); 85.3 (5.4); 102.2 (6.8); 127.1 (18.3); 130.1 (10); 146 (2.1); 239.1 (18); 257.1 (100)
			275->**257**	82.4 (23.9); 84.3 (2.8); 85.3 (8.2); 102.2 (8.2); **127.1** (100); 130.1 (10); 194.1 (3); 211.1 (3.6); 221 (6.6); 239.1 (76.2); 239.9 (4.3)
Product **4** (*in vivo* analysis of *ΔbcmB* supernatants)		MH+	289	129.9 (2.7); 196.8 (28.7); **214.8** (100)
			289->**215**	102 (2.1); 129.9 (14.5); **196.8** (100)
		[M-H]-	**287**	**212.7** (100)
Product **4** (*in vitro* reaction)	cIL + 4OH and Δ MW 288	MH+	**289.1**	130.1 (3); 197 (15.6); **215** (100)
			289->**215**	102.3 (1.1); 130.1 (16.3); **197** (100)

Supplementary Table S1. Molecular weight, m/z values and fragmentation data for bicyclomycin and all the pathway intermediates.

In vivo analyses were performed in LC-MS/MS according to the protocols described in Supplementary information. *In vitro* obtained products were analysed directly by ESI-MS/MS after their purification for NMR characterization.

2.2. Supplementary Table S2

Compound	Retention time (min)		ionisation	m/z	fragmentation	Peak area (214 nm)	Compounds identified in the shunt pathway by [2], likely to correspond to the compounds we identified
**Product c26**	21.9	**256 (cIL + 2 OH and Δ)**	MH+	**257**	210.9 (35.2); **238.9** (100)	4388	
				257->**239**	82.3 (0.5); 86.2 (1.8); 114 (1.2); 142.9 (2.9); 183 (0.7); **210.9** (100);		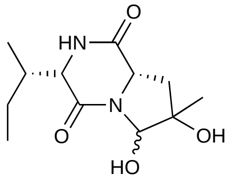
			[M-H]-	**255**	85.2 (1); 123.8 (1.3); 138.9 (7.3); 150.9 (16.7); 152.9 (1.2); 154.8 (3.4); 166.9 (14); 168.9 (10.6); **180.9** (97.4); 192.9 (86.7); 208.9 (53.1); 210.8 (65.8); 226.9 (5.9); 236.8 (100);		
				255->**181**	97 (3.7); 123.8 (100); 139.8 (3.7); 152.7 (71);		[2]: Compound 14a
**Product c27**	24.5	**258 (cIL + 2 OH)**	MH+	**259**	86.2 (3.1); 194.9 (88.9); 222.9 (33.6); 240.9 (100)	1176	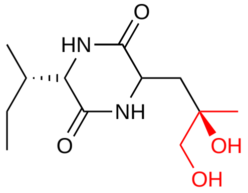
				259->**241**	82.2 (4.3); 84.2 (2.9); 86.1 (5.5); 109.9 (2.7); 114 (1.7); 126.9 (5.3); 140.8 (0.7); 167 (1.8); 194.9 (100); 222.9 (15.1)		
			[M-H]-	**257**	85.1 (1.3); 97.1 (4.5); 98 (8.5); 129 (9.7); 139 (2.6); 139.9 (38.7); 165.9 (6.3); **182.9** (100); 186.9 (4.5); 194.9 (64.1); 208.9 (15.7); 212.9 (7.8); 213.9 (13.5); 220.8 (13); 226.9 (2); 238.9 (17.1)		[2] : Compound 11
**Product c28**	25.5	**256 (cIL + 2 OH and Δ)**	MH+	**257**	86.1 (1); 98.2 (1); 126.7 (1); 142.9 (9.7); 154.9 (50.8); **182.9** (100); 210.9 (26.2); 238.9 (68.9)	290	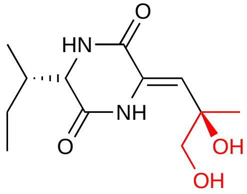
				257->**183**	86.1 (4.3); 87.1 (8.9); 98.9 (8.2); 126.9 (15.8); 138 (2.5); **154.8** (100);		

[2]: Desaturation intermediate between 11 and 12

**Product c29**	26.3	**256 (cIL + 2 OH et Δ)**	MH+	**257**	82.2 (0.7); 86.2 (1.7); 98.1 (1.1); 138.2 (0.3); 139.9 (2); 142.9 (6.9); 154.9 (0.9); 182.9 (2.4); 193 (0.6); 210.9 (100); 221 (0.7); 238.9 (22.9);	3111	
				257->**211**	69.3 (2.5); 82.2 (35.6); 84.1 (3.7); 86.2 (27.7); 98 (28); 114 (3.5); 124.9 (3.7); 125.9 (2.3); 139.9 (16.1); 142.8 (100); 154.8 (2); 182.9 (14.5);		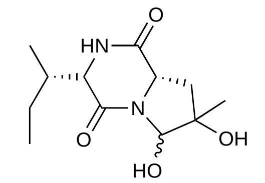
			[M-H]-	**255**	114.8 (3.5); 138.9 (4.7); 150.9 (22.5); 154.9 (4.8); 166.9 (13.2); 168.8 (8.6); 180.9 (62.5); 192.8 (88.4); 206.9 (4.4); 208.9 (46.6); 209.9 (9.7); 210.9 (51.1); 224.8 (100); 226.8 (7.4); 236.8 (85.4);		
				255->**225**	95.9 (1.3); 111.9 (3.3); 126.9 (6.4); 138.9 (3.9); 154.8 (2.4); 163.9 (3.4); 166.8 (75.9); 180.8 (5.9); 181.8 (1.5); 206.8 (100);		[2]: Compound 14b
**Product c31**	27.2	**272 (cIL + 3OH et Δ)**	MH+	**273**	86.1 (2.4); 98 (1.5); 153.9 (0.6); 170.9 (1.7); 180.8 (2.2); **198.9** (100); 208.9 (2.7); 227.9 (1.9); 236.9 (5.4); 254.9 (8.1);	2186	
				273->**199**	86.1 (83.2); 98.1 (9.6); 103 (14.8); 110 (8.1); 113.9 (6.9); 125.9 (4.7); 128 (44.9); 135.8 (10.8); 152.9 (8.5); 153.9 (33.3); 170.9 (54.5); 180.9 (100); 181.8 (7.5);		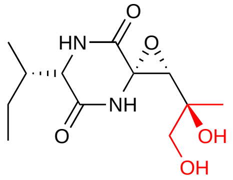
			[M-H]-	**271**	183.2 (0.6); **196.7** (100)		
				271->**197**	82.2 (2.4); 84.2 (1.4); 109.9 (1.6); 110.9 (4.2); 125.9 (8.9); 136 (1.7); 139 (1.4); 139.7 (100); 152.7 (2.3); 153.8 (31.1); 168.7 (19.9); 178.8 (6.2); 196.8 (6.9);		[2]: Compound 12
**Product c32**	29,2	**242 (cIL + OH)**	MH+	**243**	129,9 (2,2); 140,9 (3,1); 168,9 (1,8); 196,9 (4,8); 214,9 (1,2); **224,9** (100);	traces	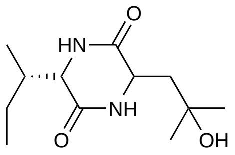

				243->**225**	69,3 (2,2); 84,3 (1,8); 85,1 (9,1); 86,2 (7,7); 112,9 (19,4); 128,9 (5,5); 129,9 (12,2); 134,9 (2); 140,9 (41,2); 151,9 (2,7); 153,9 (5,2); 156,9 (13,2); 168,8 (45,5); 179,9 (4,3); **196,9** (100);		[2]: Compound 10
**Product 6**	40.9	**cIL**	MH+	227	69.4 (3.7); 86.2 (98.3); 98 (9.3); 114 (3.8); 153.9 (24.5); 170.8 (2.3); 181.9 (100); 198.9 (93);	1359	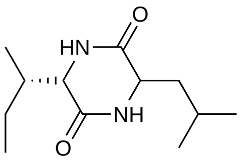 [2]: Compound 5

Supplementary Table S2: molecular weight, m/z values, fragmentation data, peak area and structure for the compounds obtained in the *bcmE* deleted mutant strain. Analyses were performed in LC-MS/MS according to the protocols described in Supplementary information. In the fragmentation data, the numbers in red correspond to the m/z values of the compound after the loss of the terminal diol of the leucyl moiety (m/z difference: 74). This diol fragment is indicated in red in the compounds.

2.3. Supplementary Table S3

Strain	Description	Plasmid	Reference or Source
***E. coli* DH5α**	*F- endA1 glnV44 thi-1 recA1 relA1 gyrA96 deoR nupG purB20* φ80d*lacZ*ΔM15 Δ(*lacZYA-argF*)U169, hsdR17($r_K^- m_K^+$), $λ^-$	-	Promega
***E. coli* BW25113pIJ790**	*F- lacI*$^+$*rrnB*$_{T14}$ Δ*lacZ*$_{WJ16}$ *hsdR*514 Δ*araBAD*$_{AH33}$ Δ*rhaBAD*$_{LD78}$ *rph-1 Δ(araB–D)567 Δ(rhaD–B)568 ΔlacZ4787(::rrnB-3) hsdR514 rph-1*	pIJ790	[3]
***E. coli* GM119**	*F- dam-3 dcm-6 metBI gaIK2 gaIT22 lacY1 tsx-78 supE44 (thi-1 tonA3I mtl-I)?*	-	[4]
***E. coli* ET12567pUZ8002**	*F- dam13::Tn9 dcm-6 hsdM hsdR zjj-202::Tn10 recF143 galK2 galT22 ara-14 lacY1 xyl-5 leuB6 thi-1 tonA31 rpsL136 hisG4 tsx-78 mtl-1 glnV44*	pUZ8002	[5]
***E. coli* BL21-AI**	F^-*ompT* *hsd*S_B (r_B^- m_B^-) *gal dcm* *ara*B::T7RNAP-*tet*A		Thermo Fisher Scientific
***S. cinnamoneus* DSM 41675**	Wild-type producer of bicyclomycin	-	Leibniz Institute DSMZ – German Collection of Microorganisms and Cell Cultures
S. cinnamoneus* Δ*bcm	Δ*bcmH-bcmG::aphII*	-	This study
S. coelicolor M1154	*Δact*, *Δred, Δcpk, Δcda, rpoB* [C1298T], *rpsL* [A262G]		[6]
S. lividans TK24	SLP2$^-$, SLP3$^-$, *strR*		[7]

Supplementary Table S3 Bacterial strains used in this study

2.4. Supplementary Table S4

Plasmid	Description	Reference or Source
**pET15b**	*E. coli* replicative vector for expression of His-Tagged protein, Amp^r	Novagen / Merck-Millipore
**pUC18**	*E. coli* cloning vector, Amp^r	Thermo Fischer Scientific
**pHP45Ω**	Source of Ω*aadA* cassette, Amp^r, $Spec^r$	[8]
**pOSV400**	*Streptomyces* suicide vector for gene deletion, $Apra^r$, Hyg^r	[9]
**pRT801**	*Streptomyces* ΦBT1 integrative vector, $Apra^r$	[10]
**pOSV408**	*Streptomyces* ΦBT1 integrative vector, Kan^r	[11]
**pOSV668**	*Streptomyces* ΦBT1 integrative vector, $Apra^r$, $Spec^r$	this study
**pOSV806**	*Streptomyces* ΦC31 integrative vector, Hyg^r	[12]
**pJWm07**	pOSV400 containing the *aphII* gene flanked with the upstream and downstream region of *bcm* cluster, for the deletion of the *bcm* cluster in *S. cinnamoneus*, Hyg^r, Kan^r	this study
**pJWc13**	pUC18 containing *orf1-bcmA'*	this study
**pJWc14**	pUC18 containing *bcmA'-CBP*	this study
**pJWe14**	pOSV668 containing the *bcm* cluster	this study
**pJWe20**	pOSV668 containing the *bcmA* gene with its native promoter	this study
**pJWe21**	pOSV806 containing the *bcmH* gene with its native promoter	this study
**pMC1**	pJWe14 with *bcmB::ΩaadA*	this study
**pMC2**	pJWe14 with in frame deletion in *bcmB*	this study
**pMC3**	pJWe14 with *bcmC::ΩaadA*	this study
**pMC4**	pJWe14 with in frame deletion in *bcmC*	this study
**pMC5**	pJWe14 with *bcmD::ΩaadA*	this study
**pMC6**	pJWe14 with in frame deletion in *bcmD*	this study
**pMC7**	pJWe14 with *bcmE::ΩaadA*	this study
**pMC8**	pJWe14 with in frame deletion in *bcmE*	this study
**pMC9**	pJWe14 with *bcmF::ΩaadA*	this study
**pMC10**	pJWe14 with in frame deletion in *bcmF*	this study
**pMC11**	pJWe14 with *bcmG::ΩaadA*	this study
**pMC12**	pJWe14 with in frame deletion in *bcmG*	this study
**pMC13**	pJWe14 with *bcmA::ΩaadA*	this study
**pMC14**	pJWe14 with in frame deletion in *bcmA*	this study
**pMC15**	pJWe14 with *bcmH::ΩaadA*	this study
**pMC16**	pJWe14 with in frame deletion in *bcmH*	this study
**pJDCyp**	pET15b containing the *bcmD* gene	this study
**pJDOG1**	pET15b containing the *bcmB* gene	this study
**pJDOG2**	pET15b containing the *bcmC* gene	this study
**pJDOG3**	pET15b containing the *bcmE* gene	this study
**pJDOG4**	pET15b containing the *bcmF* gene	this study
**pJDOG5**	pET15b containing the *bcmG* gene	this study

Supplementary Table S4. Plasmids used in this study

Amp^r: ampicillin resistance

$Apra^r$: apramycin resistance

Hyg^r: hygromycin resistance

Kan^r: kanamycin resistance

$Spec^r$: spectinomycin resistance

2.5. Supplementary Table S5

Primer	Séquence	Use
**JD1**	GGGAATTCCATATGACCATGTCCCGTGCACC	Cloning of *bcmB* into pET15b
**JD2**	CCGCTCGAGTCAGTGCTCACGTCGCTTGT	Cloning of *bcmB* into pET15b
**JD3**	GGGAATTCCATATGAGCACTGAGACGCTGCG	Cloning of *bcmC* into pET15b
**JD4**	CCGCTCGAGTCATGCCCTCACCCCGTCTT	Cloning of *bcmC* into pET15b
**JD5**	GGGAATTCCATATGACCGCGCCCGCCCACCC	Cloning of *bcmD* into pET15b
**JD6**	CCGCTCGAGTCAGTTCTGCTCCTTCGTGC	Cloning of *bcmD* into pET15b
**JD7**	GGGAATTCCATATGGCGTCACCCGATTCCGC	Cloning of *bcmE* into pET15b
**JD8**	CCGCTCGAGTCAGAGCGCACCGCTGTCGT	Cloning of *bcmE* into pET15b
**JD9**	GGGAATTCCATATGACGACGGTCGTCGACAA	Cloning of *bcmF* into pET15b
**JD10**	CCGCTCGAGTCAAATGCGGGAGTGGAACTCGTA	Cloning of *bcmF* into pET15b
**JD11**	GGGAATTCCATATGAGCACGGCACAGGGATA	Cloning of *bcmG* into pET15b
**JD12**	CCGCTCGAGTCAGTACAGGCCGGCGGTGT	Cloning of *bcmG* into pET15b
**JWc8**	CCCAAGCTTTCATCGCCCTTTCAGGTGGA	Cloning of *bcmA* into pOSV668
**JWc9**	CCCAAGCTTAGCAGCAGGAGACACGATGG	Amplification and assembly of *bcm* cluster into pUC18
**JWc10**	AAGAACCCGCCGGAATTCAC	Amplification and assembly of *bcm* cluster into pUC18/Deletion of *bcm* cluster
**JWc11**	ACACCACTCCGCCTTCTTAC	Amplification and assembly of *bcm* cluster into pUC18
**JWc12**	TGAACGATCCGGGATACACC	Amplification and assembly of *bcm* cluster into pUC18
**JWc13**	CCGCGACTACCACGGCAAAC	Amplification and assembly of *bcm* cluster into pUC18
**JWc14**	GGGCGTCGTCTCGATGTAGG	Amplification and assembly of *bcm* cluster into pUC18
**JWc15**	ACGTGATCCGGCGCTACTTC	Amplification and assembly of *bcm* cluster into pUC18
**JWc16**	CCTGTGCCGTGCTCATATGC	Amplification and assembly of *bcm* cluster into pUC18
**JWc17**	CGGCACCTTGCACGAGTACC	Amplification and assembly of *bcm* cluster into pUC18
**JWc18**	CCCAAGCTTCCTTCGCCAAGCCCACCTCC	Amplification and assembly of *bcm* cluster into pUC18
**JWc29**	CCCAAGCTTAGGCGTCCAGTTGGATGCAG	Cloning of *bcmA* into pOSV668
**JWc30**	CCAATGCATCACCACTCCGCCTTCTTACC	Cloning of *bcmH* into pOSV806
**JWc31**	GGACTAGTGGCTCACTCCGAGGAAACAG	Cloning of *bcmH* into pOSV806
**JWm19**	GGATCTTCACCTAGATCCTT	Amplification of *aphII* gene
**JWm20**	CCGGAATTCGGCAAGACCGATCCCCGGGG	Amplification of *aphII* gene
**JWm23**	CCCAAGCTTCACCGGGACCCGTAAGTGAG	Deletion of *bcm* cluster
**JWm24**	CCGGAATTCAGACGCGGATCGGCCGCTCG	Deletion of *bcm* cluster
**JWm25**	GACTAGTGGCGCGGAGGTGCGGGTATG	Deletion of *bcm* cluster
**JW-RTc5**	CCAAGGCCGTTTCCTCCAAG	RT-PCR analysis of *rpoD* gene
**JW-RTc6**	TCCTCGGCGATGATCTCCAG	RT-PCR analysis of *rpoD* gene
**JW-RT7**	CAGCGCCATGACGTTCACCG	RT-PCR analysis of *bcmH* gene
**JW-RT8**	TCCTGGCGCGTCATCTTCTG	RT-PCR analysis of *bcmH* gene
**JW-RT9**	CGCTACAAGGCGGAGATAGG	RT-PCR analysis of *bcmA* gene ; pJWm07 Sequencing verification
**JW-RT10**	TGAACGATCCGGGATACACC	RT-PCR analysis of *bcmA* gene ; pJWm07 Sequencing verification
**JW-RT11**	CGTCTTCCTCCTGGAGATAC	RT-PCR analysis of *bcmB* gene
**JW-RT12**	TCGACAGACCGAAGTTGATG	RT-PCR analysis of *bcmB* gene ; Verification of deletion of *bcm* cluster
**JW-RT13**	AGCACTTCCAGACCGAACAC	RT-PCR analysis of *bcmC* gene
**JW-RT14**	AAGTCGCGCAGGAAGTCCTC	RT-PCR analysis of *bcmC* gene
**JW-RT15**	TTCGGCGACTTCATGGGCTC	RT-PCR analysis of *bcmD* gene
**JW-RT16**	TCACCGTGCCCAGTTCCTTG	RT-PCR analysis of *bcmD* gene
**JW-RT17**	GCGACTACCAGGGCTACTTC	RT-PCR analysis of *bcmE* gene
**JW-RT18**	AGACGACGCCGTGCACATTG	RT-PCR analysis of *bcmE* gene

**JW-RT19**	TCCTGGAGCGGATGAAGGAC	RT-PCR analysis of *bcmF* gene
**JW-RT20**	AGCCCTGGTACTCGTGCAAG	RT-PCR analysis of *bcmF* gene
**JW-RT21**	GAGCAGTTCTTCCTGGAGAG	RT-PCR analysis of *bcmG* gene
**JW-RT22**	AGGCTGCTGTCGACGAAGAG	RT-PCR analysis of *bcmG* gene
**JW-RT23**	TCGTCCGGTACGGACATCTC	RT-PCR analysis of *orf -1* gene
**JW-RT24**	CCCGTGGTGAAGCGTTTCTG	RT-PCR analysis of *orf -1* gene
**JW-RT25**	AGAGAACGCCCGGCAGATAC	RT-PCR analysis of *orf -2* gene
**JW-RT26**	AGGTCGGAGCGGCATATGTC	RT-PCR analysis of *orf -2* gene
**JW-RT27**	CGTCGGAGCAGGTGTAGAAG	RT-PCR analysis of *orf +1* gene
**JW-RT28**	AGCCGCTCTACGACTGGAAC	RT-PCR analysis of *orf +1* gene
**JW-RT29**	CATGTCGCCGCTGAAAGAGG	RT-PCR analysis of *orf +2* gene
**JW-RT30**	CCGGCGTGAAGCACTTCAAC	RT-PCR analysis of *orf +2* gene
**JWseq6**	CCCTTTTTTGGCCTTGAAAT	Sequencing and verification of pJWe14 and derivatives
**JWseq7**	GAATTGTGAGCGGATAACAA	Sequencing and verification of constructions (pJWe14 and derivatives, derivatives of pET15b)
**MC1**	ATCGATGCGAGGCAAGCTTATCGATG	Amplification of *ΩaadA* cassette
**MC2**	ATCGATTATCACGAGGCCCTTTCGTC	Amplification of *ΩaadA* cassette
**MC3**	TGAAAGGGCGATGACCATGTCCCGTGCACCCGGC AACACGATCGATGCGAGGCAAGCTTA	Deletion of *bcmB* by PCR targeting
**MC4**	CGTGGTCGTTCTCGTCGATGAGTTCCCGGGAGCC GCAGACATCGATTATCACGAGGCCCT	Deletion of *bcmB* by PCR targeting
**MC5**	GACAAGCGACGTGAGCACTGAGACGCTGCGCCTC CAGAAGATCGATGCGAGGCAAGCTTA	Deletion of *bcmC* by PCR targeting
**MC6**	TCCCGAAGTCGGCGTAGCCGTCGCCCCACGTCCG CTCGTTATCGATTATCACGAGGCCCT	Deletion of *bcmC* by PCR targeting
**MC7**	GGGTGAGGGCATGACCGCGCCCGCCCACCCGCCG GCCTGTATCGATGCGAGGCAAGCTTA	Deletion of *bcmD* by PCR targeting
**MC8**	CGGGCCGGGCGGGCCGGGGCATGGCGGCGTGCCT CTTCAGATCGATTATCACGAGGCCCT	Deletion of *bcmD* by PCR targeting
**MC9**	AGCAGAACTGATGGCGTCACCCGATTCCGCCACC CTCCGGATCGATGCGAGGCAAGCTTA	Deletion of *bcmE* by PCR targeting
**MC10**	ACTCGGCCACCGACTGGAGGGGCCGGGGCGTGCC GTTCTCATCGATTATCACGAGGCCCT	Deletion of *bcmE* by PCR targeting
**MC11**	AAGGACTGCGATGACGACGGTCGTCGACAACGAA GGACACATCGATGCGAGGCAAGCTTA	Deletion of *bcmF* by PCR targeting
**MC12**	AGAAGTCGCGGAAGCCCTGGTACTCGTGCAAGGT GCCGTCATCGATTATCACGAGGCCCT	Deletion of *bcmF* by PCR targeting
**MC13**	CCCGCATATGAGCACGGCACAGGGATACGGCTGG CAGACGATCGATGCGAGGCAAGCTTA	Deletion of *bcmG* by PCR targeting
**MC14**	CGAGGAACGTGCCGAAGTTCGTCTCCAGGCGGAG ACCGGTATCGATTATCACGAGGCCCT	Deletion of *bcmG* by PCR targeting
**MCc1**	GGTTGGCTTTATGTCGCTAGAAGCGCAGCTGATG GAGCCTATCGATGCGAGGCAAGCTTA	Deletion of *bcmA* by PCR targeting
**MCc2**	CGGGGGCTCCCGGGTGTTTTCCCTGAGCTATTTC CGAGAGATCGATTATCACGAGGCCCT	Deletion of *bcmA* by PCR targeting
**MC-Cl1**	GAGATCACCAAGGTAGTCGG	Sequencing and verification of pMC1 to pMC15 (odd number)
**MCm1**	GGCCCGGGGCCTACCTCCGGCCCGTCCGGGCCAT CGCCCTATCGATGCGAGGCAAGCTTA	Deletion of *bcmH* by PCR targeting
**MCm2**	GGCCCTGCCGAGCATCGGTCACGACCTCGGCGCC TCGACCATCGATTATCACGAGGCCCT	Deletion of *bcmH* by PCR targeting
**MCpseq1**	TGGCACCCAGCCTGCGCGAG	Sequencing and verification of pMC1 to pMC15 (odd number)
**MCpseq2**	ATAAGCCCTACACAAATTGG	Sequencing and verification of pMC1 to pMC11 (odd number)
**MCpseq3**	ACCGGGCGGAAGAACTCGCG	Sequencing and verification of pMC1
**MCpseq4**	TGCACCGCGTCGCCCGGCAG	Sequencing and verification of pMC3
**MCpseq5**	CCCGCCCGGACACGGGACAG	Sequencing and verification of pMC3
**MCpseq6**	CCGGAGTCCGCCGACCGCTT	Sequencing and verification of pMC5
**MCpseq7**	CCGGCCGGCTGTTCTCCGTG	Sequencing and verification of pMC7
**MCpseq8**	CGTTGTCGACGACCGTCGTC	Sequencing and verification of pMC7
**MCpseq9**	GACCGAACGCCTCGACCGAC	Sequencing and verification of pMC9
**MCpseq10**	AACCTGGGTGACGCGTTCCG	Sequencing and verification of pMC11

Supplementary Table S5. Primers used in this study

Restriction sites added and used for cloning are underlined.

2.6. Supplementary Table S6

Dominant rotamers for the leucyl chain of product **5** along with their relative configurations. NOE correlations are classified into strong (s), medium (m) and weak (w) intensities (abs = absent).

Fragment	Rotamer	$^{3}J_{\text{H-H}}$ (Hz)	Selected $^{2,3}J_{\text{CH}}$ (Hz)	Key NOEs
C_α–C_β	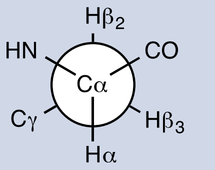	$^{3}J_{\text{H}\beta2\text{H}\alpha} = 9.6$<br>$^{3}J_{\text{H}\beta3\text{H}\alpha} = 2.8$	$^{3}J_{\text{H}\beta2\text{CO}} = 1.9$<br>$^{3}J_{\text{H}\beta3\text{CO}} = 2.5$<br>$^{3}J_{\text{H}\alpha\text{C}\gamma} = 1.5$	HN-$H_{\beta2}$ (m)<br>*HN-$H_{\beta3}$ abs*<br>HN-$HO_{C\gamma}$ (m)<br>*H_α-$H_{\beta2}$ abs*<br>H_α-$H_{\beta3}$ (s)
C_β–C_γ	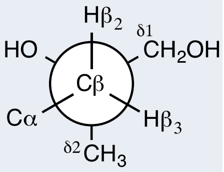		$^{2}J_{\text{H}\beta2\text{C}\gamma} = 4.6$<br>$^{2}J_{\text{H}\beta3\text{C}\gamma} = 3.0$<br>$^{3}J_{\text{H}\beta2\text{C}\delta1} = 2.2$<br>$^{3}J_{\text{H}\beta3\text{C}\delta1} = 1.9$<br>$^{3}J_{\text{H}\beta2\text{C}\delta2} = 5.0$<br>$^{3}J_{\text{H}\beta3\text{C}\delta2} = 2.0$	H_α-$Me_{\delta2}$ (s)<br>$H_{\beta3}$-$Me_{\delta2}$ (m)<br>H_α-OH (m)<br>$H_{\beta2}$-OH (m)

2.7. Supplementary Table S7

Dominant rotamers for the leucyl chain of product **4** along with their relative configurations. NOE correlations are classified into strong (s), medium (m) and weak (w) intensities.

Fragment	Rotamer	Selected $^{2,3}J_{\text{CH}}$ (Hz)	Key NOEs
C_α–C_β	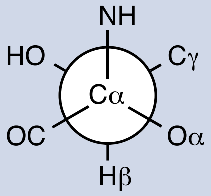	$^{2}J_{\text{H}\beta\text{C}\alpha} = 4.2$<br>$^{3}J_{\text{H}\beta\text{CO}} = 2.1$	HN-H_β (m)<br>HN-$H_{\delta12}$ (m)<br>HN-$H_{\delta13}$ (m)<br>HN-$Me_{\delta2}$ (w)<br>HN-$HO_{C\gamma}$ (w)
C_β–C_γ	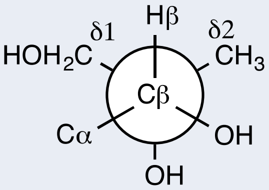	$^{2}J_{\text{H}\beta\text{C}\gamma} \approx 0$<br>$^{3}J_{\text{H}\beta\text{C}\delta1} = 1.2$<br>$^{3}J_{\text{H}\beta\text{C}\delta2} = 1.5$	H_β-$Me_{\delta2}$ (s)<br>H_β-$H_{\delta12}$ (s)<br>H_β-$H_{\delta13}$ (s)<br>$HO_{C\beta}$-$Me_{\delta2}$ (m)

3. Supplementary methods

3.1. Deletion of the *bcm* cluster

The DNA region upstream of the *bcm* cluster was amplified by PCR with *S. cinnamoneus* genomic DNA as a template and oligonucleotides JWm23 and JWc10. The resulting PCR fragment was isolated as a *Hin*dIII/*Eco*RI fragment. The *aphII* kanamycin resistance gene was amplified using pOSV408 as template and oligonucleotides JWm19 and JWm20. The *aph*II gene was purified as an *Eco*RI fragment. The DNA region downstream of the *bcm* cluster was amplified by PCR with *S. cinnamoneus* genomic DNA as template and oligonucleotides JWm24 and JWc25. This region was purified as an *Eco*RI/*Bcu*I fragment. These three fragments were cloned together into *Hin*dIII/*Bcu*I-digested suicide vector pOSV400 to create pJWm07. Effective cloning of the fragments in pJWm07 was controlled by sequencing.

Sequencing of pJWm07 revealed the presence of two mutations in the insert (Supplementary Figure S24). The first one was a point mutation G1021T in the intergenic region upstream of *orf-2*. The second one was a deletion of nucleotides 6073 to 6374. This deletion included the last bp of *orf+2*, the intergenic region between *orf+2* and *orf+1* and the first 43 bp of *orf+1*. For unknown reasons, we were unable to obtain the PCR product of the downstream region without this deletion. As these two mutations are in genes which are not involved in bicyclomycin biosynthesis, did not impair our bicyclomycin BCG characterization, we nevertheless used this mutated plasmid pJWm07 to construct the Δ*bcm* strain.

pJWm07 was transferred to *S. cinnamoneus* by conjugation according to [13]. Exconjugants were selected for kanamycin resistance. Hygromycin-sensitive and kanamycin-resistant clones were then screened. Their genomic DNA was extracted and the replacement of the cluster by the kanamycin resistance cassette was verified by PCR using oligonucleotides JW-RT9 and JW-RT12.

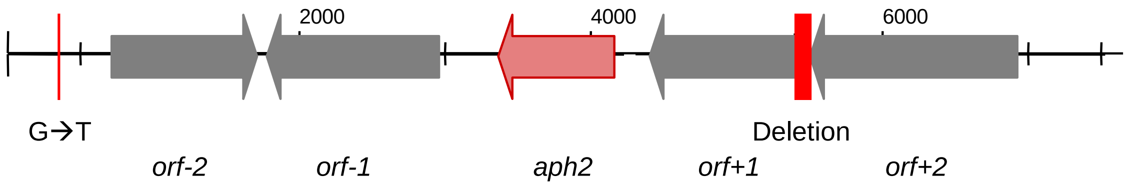

Supplementary Figure S24. The genetic organization of the Δ*bcm* mutant strain. Unwanted mutations induced during the strain construction are marked in red.

3.2. Plasmid-born expression of *bcm* genes

3.2.1. Construction of pJWe14, harbouring the entire *bcm* cluster

Plasmid pOSV668 was first constructed by cloning the spectinomycin resistance cassette *ΩaadA* from pHP45Ω into pRT801 at the *Bam*HI site.

The *bcm* cluster was amplified as five independent fragments by PCR with the *S. cinnamoneus* genomic DNA as a template.

Fragment I was amplified with oligonucleotides JWc9 and JWc10, then purified as an *Hin*dIII/*Eco*RI fragment. Fragment II was amplified with oligonucleotides JWc11 and JWc12, then purified as an *Eco*RI/*Xba*I fragment. Fragments I and II were cloned together into *Hin*dIII/*Xba*I-digested pUC18 to create plasmid pJWc13.

Fragment III was amplified with oligonucleotides JWc13 and JWc14 and purified as a *Xba*I/*Mls*I fragment. Fragment IV was amplified with oligonucleotides JWc15 and JWc16, purified as a *Mls*I/*Nde*I fragment. Fragment V was amplified with oligonucleotides JWc17 and JWc18, then purified as a *Nde*I/*Hin*dIII fragment. Fragments III, IV and V were cloned together into *Hin*dIII/*Xba*I-digested pUC18 to create plasmid pJWc14.

The integrity of inserts in pJWc13 and pJWc14 was verified by sequencing.

Last, the whole cluster was assembled by ligating fragments I-II, excised by *Hin*dIII/*Xba*I from pJWc13, fragments III-V, excised by *Hin*dIII/*Xba*I from pJWc14, into *Hin*dIII-digested pOSV668, creating pJWe14. The integrity of pJWe14 was verified by restriction.

3.2.2. Deletion of individual *bcm* genes on pJWe14

All the genes of the *bcm* cluster were individually deleted by a PCR-targeting approach. For this purpose, the Ω*aadA* cassette was first amplified using pHP45Ω as template and MC1 and MC2 as primers. MC1 and MC2 added *Bsu*15I (*Cla*I) restriction site at each end of the amplicon. The obtained amplicon was isolated on agarose gel and used as template for a set of new PCR reactions with the primer couples MC3/MC4, MC5/MC6, MC7/MC8, MC9/MC10, MC11/MC12, MC13/MC14, MCc1/MCc2 and MCm1/MCm2. These primers present 40 bp-long identical regions with the genes *bcmB*, *bcmC*, *bcmD*, *bcmE*, *bcmF*, *bcmG*, *bcmA* and *bcmH* respectively. They were designed in order to replace, after homologous recombination, most of the coding sequence of each gene with the spectinomycin resistance cassette Ω*aadA*, leaving 30 base pairs at the beginning of the coding sequence and 90 at the end. The PCR products were used in a PCR-targeting procedure on pJWe14, following the protocol previously described [3], yielding the plasmids pMC1, pMC3, pMC5, pMC7, pMC9, pMC11, pMC13 and pMC15. Those constructs were introduced in *E. coli* GM119, a non-methylating strain; their integrity was verified by sequencing. The Ω*aadA* cassette was finally

removed by digestion by *Bsu*15I followed by plasmid self-circularization, yielding the plasmids pMC2, pMC4, pMC6, pMC8, pMC10, pMC12, pMC14 and pMC16 (Supplementary Table S4).

3.2.3. Construction of pJWe20 for the expression of *bcmA* under the control of its native promoter

PCR amplification using oligonucleotides JWc29, JWc8 and the genomic DNA of *S. cinnamoneus* as a template was carried out. The PCR product was purified as a *Hin*dIII fragment and cloned into *Hin*dIII-digested pOSV668, creating pJWe20. The integrity of the insert was verified by sequencing.

3.2.4. Construction of pJWe21 for the expression of *bcmH* under the control of its native promoter

PCR amplification using oligonucleotides JWc30, JWc31 and the genomic DNA of *S. cinnamoneus* as template was carried out. The PCR product was purified as a *Mph*1103I/*Bcu*I fragment and cloned into pOSV806 digested by the same enzymes, creating pJWe21. The integrity of the insert was verified by sequencing.

3.2.5. Cloning of *bcm* tailoring genes into the pET15b vector

The *bcmB, bcmC, bcmD, bcmE, bcmF* and *bcmG* genes were independently amplified by PCR, with the primer couples JD1/JD2, JD3/JD4, JD5/JD6, JD7/JD8, JD9/JD10 and JD11/JD12, respectively. Forward primers introduced a *Nde*I restriction site and Reverse primers a *Xho*I site. PCR products were digested by *Nde*I and *Xho*I, ligated into *Nde*I/*Xho*I-digested pET15b, yielding plasmids pJDOG1, pJDOG2, pJDCyp, pJDOG3, pJDOG4 and pJDOG5, respectively. The integrity of each insert was verified by sequencing.

3.3. *In vitro* characterization of the bicyclomycin tailoring pathway

3.3.1. Protein production and purification

E. coli BL21-AI competent cells were transformed with either pJDOG1, pJDOG2, pJDCyp, pJDOG3, pJDOG4 or pJDOG5. For each of the resulting strains, an overnight culture was used to inoculate 1 l of liquid LB medium (initial OD_{600} 0.05). For the strain transformed with pJDCyp, 100 mg/l aminolevulinic acid was added to the medium. Cultures were grown at 37 °C under orbital agitation (200 rpm) up to OD_{600} 0.3-0.4 and then cooled to 20 °C. Target gene expression was induced by the addition of 1 mM IPTG and 0.2% L-arabinose and cultures were further grown overnight at 20 °C under orbital agitation (200 rpm). For each protein of interest, cells were harvested by

centrifugation (8,000 g, 4°C, 20 min), resuspended in 30 ml buffer A (50 mM HEPES pH 7.5, 0.5 M NaCl, 20 mM imidazole, 5% glycerol, 1 mM DTT and 1 mM PMSF) and disrupted with an Eaton press. $MgCl_2$ and benzonase were added to a final concentration of 2 µM and 0.1 U/ml, respectively, and lysates were incubated at 4 °C for 45 min under gentle agitation. After centrifugation (20,000 g, 4 °C, 30 min), the supernatant was loaded onto a 5 ml HisTrap HP column (GE Healthcare) using a flow rate of 1 ml/min (Äkta Purifier FPLC, GE Healthcare) and the column was extensively washed with buffer A (60 ml). Proteins were eluted with a 10 column volumes linear gradient of imidazole (20 to 160 mM) in buffer A, followed by an extensive isocratic step (10 column volumes) with 160 mM imidazole in buffer A. Fractions containing the target polypeptide were identified by their absorbance at 280 nm, pooled and applied onto a HiTrap Desalting column (Äkta Purifier, GE Healthcare). The protein of interest was recovered in buffer C (20 mM HEPES pH 7.5, 0.1 M NaCl, 1 mM DTT, 1 mM PMSF), concentrated with Amicon Ultra-4 or Ultra-15 10,000 NMWL filters (EMD Millipore) and analysed by SDS-PAGE. Glycerol was added to a final concentration of 10% and the purified protein was stored at -80 °C.

3.3.2. Enzymatic assays

Enzymatic assays were performed at 30 °C in a volume of 1 ml.

For 2-oxoglutarate/iron dependent dioxygenase assays, the reaction buffer contained 50 mM HEPES pH 7.5, 100 µM DTT, 50 µM $FeSO_4$, 2 mM sodium L-ascorbate, 2 mM disodium 2-oxoglutarate, 100 mg/l bovine liver catalase and 200 µM diketopiperazine substrate cIL (**2**), unless otherwise stated; **2** being poorly soluble in water, reaction mixtures with this compound contained 2% DMSO.

Reactions contained 1 µM of each of the purified proteins to be tested. Assays were made with one single protein, or with combinations of two to five different proteins.

Enzymatic assays with the putative cytochrome P450 monooxygenase BcmD were incubated for 24 h in reaction mixtures containing 51 mM potassium phosphate pH 7.5, 15 mM Tris-HCl pH 7.5, 10 µM *Spinacia oleracea* ferredoxin, 10^{-3} U/ml *Spinacia oleracea* ferredoxin reductase, 1 mM NADPH, 100 µM of the purified **4** (see below) and 1 µM BcmD.

For all the assays, 100 µl aliquots were collected at times 0, 3, 10 and 60 min. Reactions were stopped on ice by acidification with formic acid (2% final concentration).

3.3.3. HPLC and LC-MS analysis of the enzymatic assays

Enzymatic assays were analysed as described by [14] for CDPS 1-47 with an Atlantis dC18 column and a VWR/Hitachi Elite LaChrom instrument. Interesting peaks were recuperated from the flow-through and injected to an Esquire HCT ion trap mass spectrometer (Bruker) set in positive and negative modes.

3.3.4. Production and purification of intermediates of the bicyclomycin pathway

For NMR analyses, the production of all compounds was scaled-up as follows.

For **7,** incubation was performed for 5 h in 20 ml reaction mixture containing 1 mM **2** (cIL) (10 % DMSO final concentration) and BcmE (5 µM).

For **6**, incubation was performed for 5 h in 40 ml reaction mixture containing 0,5 mM of **2** (cIL) (5 % DMSO final concentration), BcmE (5 µM) and BcmC (5 µM).

For **5**, incubation was performed for 5 h in 2.25 ml reaction mixture containing 2 mM of the purified **6** as the substrate and 1 µM BcmG.

For **4**, incubation was performed for 2 h in 3 ml reaction mixture containing 2 mM of the purified **5** as the substrate, 6 mM 2-oxoglutarate and BcmB (5 µM).

For **3**, incubation was performed for 24 h in 4 ml reaction mixture containing 0.5 mM of the purified **4** as the substrate and 1 µM BcmD.

All the reaction mixtures were incubated in 50 ml Falcon tubes, with no more than 5 ml per tube at 30 °C under gentle orbital agitation (60 rpm).

Products **5**, **6** and **7** were purified on a LiChroCART 250 x 10 Purospher STAR RP-18e (5 µm) column (Merck), using a VWR/Hitachi Elite LaPrepΣ instrument. Samples containing DMSO were loaded (5 ml maximum at once) and washed with 100% solvent A (0.1% HCOOH in water) for 30 minutes, then eluted by gradients starting with 100% solvent A and 0% solvent B (0.1% HCOOH in 10 % water, 90 % acetonitrile). The flow was at 4.75 ml/min.

The exact gradients were set as follows:

For the purification of **7**, the product of the reaction catalysed by BcmE with cIL (c **2**) alone as a substrate, the linear gradient was set as to reach a 30% solvent B concentration in 30 minutes.

For the purification of compound **6**, the product of the reaction catalysed by BcmE and BcmC with cIL (**2**) alone as a substrate, a first linear gradient was set to reach 10 % of solvent B in 5 minutes, followed by a second linear gradient to reach 20% solvent B in 20 minutes.

For the purification of compound **5**, as the reaction mixture did not contain DMSO, it was loaded and directly washed with 100% solvent A during 5 minutes. Compound **5** was then eluted with a linear gradient set to reach 20% solvent B in 20 minutes.

The purification of **4**, the product of the reaction catalyzed by BcmB with **5** as the substrate, was performed on an Hypercarb 150 x 10 (5 µM) column (Thermo Fisher Scientific). The reaction mixture was loaded and washed with 100 % solvent A for 5 minutes, then eluted with a linear gradient reaching 25% solvent B in 25 minutes. The collected peak contained a mixture of **4** and **5**,

was lyophilized, dissolved in water and purified on a LiChroCART 250 x 10 Purospher STAR RP-18e (5 µm) column (Merck) using the same loading and elution conditions as before.

The purification of **3**, the product of the reaction catalysed by BcmD with **4** as the substrate, was performed on an ACE Excel 3 C18-PFP (150 x 4.6 mm) column (Advanced Chromatography Technologies). The flow was at 0.6 ml/min and the gradient as the same as for purification of **4**.

All the products obtained were lyophilized and their identities were confirmed by NMR and mass spectrometry analyses.

3.4. NMR Analyses

Samples of compounds cIL (**2**) (10.6 mM), **7** (5 mM), **6** (20.9 mM), **5** (4.1 mM) and **4** (< 1 mM) were prepared in DMSO-d_6 (Eurisotop, Saint-Aubin, France) in 3 or 5 mm NMR tubes (corresponding volumes 0.2 or 0.5 mL, respectively). NMR experiments were recorded on a 500 MHz Bruker Avance III spectrometer equipped with a 5-mm inverse TCI cryoprobe incorporating a Z-gradient coil. Spectra were recorded at 298.6 K. All data were processed and analyzed with Bruker TopSpin 3.2 program. ^{1}H and ^{13}C resonances were assigned via the analysis of one-dimensional ^{1}H, one-dimensional ^{13}C DEPTQ (Distortionless Enhancement by Polarization Transfer), two-dimensional ^{1}H-^{1}H COSY, two-dimensional ^{1}H-^{1}H TOCSY (Total Correlation Spectroscopy, mixing time of 66 ms), two-dimensional ^{1}H-^{1}H NOESY (Nuclear Overhauser Effect Spectroscopy, mixing time of 1.5 s), two-dimensional ^{1}H-^{13}C HSQC (Heteronuclear Single Quantum Correlation), two-dimensional ^{1}H-^{13}C HMBC (Heteronuclear Multiple-Bond Correlation). ^{1}H and ^{13}C chemical shifts were referenced to the residual protiated DMSO solvent signal (δ ^{1}H 2.50 ppm) or deuterated DMSO solvent (δ ^{13}C 39.5 ppm), respectively. Homonuclear $J_{H,H}$ couplings were measured on 1D ^{1}H spectra processed with Lorentz-Gauss apodization. 1D selective irradiations were applied to extract $J_{H,H}$ couplings for the most complex multiplets. Heteronuclear $^{n}J_{H,C}$ couplings were measured using 2D ^{1}H-^{13}C IPAP-HSQMBC (In-phase Anti-Phase Heteronuclear Single Quantum Multiple Bond Correlation) experiments, with an evolution delay $\Delta = 1 / (2 \times {}^{n}J_{CH})$ optimized for $^{n}J_{H,C}$ couplings of 8 Hz or 6 Hz [15]. Stereochemical analysis was based on NOEs, $^{3}J_{H,H}$ and $^{2,3}J_{C,H}$ coupling constants. Homonuclear vicinal coupling constants ($^{3}J_{H,H}$) and heteronuclear coupling constants ($^{3}J_{C,H}$) depend on dihedral angles via Karplus relationships [16–18]. $^{2}J_{C,H}$ coupling constants were also useful for the analysis of oxygen-substituted two-carbon fragments as they depend on the dihedral angle between the proton and ^{13}C-attached oxygen: $^{2}J_{C,H}$ coupling constant is large (typically 4–7 Hz) when an oxygen substituent on a carbon atom is *gauche* to the

nearby proton, whereas $^2J_{C,H}$ value is smaller (typically 0–3 Hz) when the proton and ^{13}C-attached oxygen have *anti* relationship.

3.5. Analysis of transcription by RT-PCR

Total RNA was isolated using the NucleoSpin RNA and NucleoSpin RNA/DNA Buffer Set kits (Macherey-Nagel). The RNA samples were treated with RNAse-free DNAse (Ambion) followed by purification using the NucleoSpin RNA Clean-up kit (Macherey-Nagel). The absence of DNA contamination was verified by a 30-cycle PCR with primer couples for all the genes analysed. Reverse transcription reaction was performed with RevertAid First Strand cDNA Synthesis (Thermo Fischer Scientific) using the Random Hexamer primer and the protocol for GC-rich templates. The generated cDNA served for a PCR amplification (denaturation: 95°C, 30 s, hybridation: 60°C, 30 s, elongation: 72°C, 30 s, 28 cycles). The primers used for this analysis are listed in Supplementary Table S5.

4. SI references

1. Bradley, E. L. *et al.* The biosynthesis of the *Streptomyces* antibiotic bicyclomycin. *Tetrahedron Letters* **37**, 6935–6938 (1996).
2. Meng, S. *et al.* A six-oxidase cascade for tandem C-H Bond activation revealed by reconstitution of bicyclomycin biosynthesis. *Angew. Chem. Int. Ed. Engl.* **57**, 719–723 (2018).
3. Gust, B., Challis, G. L., Fowler, K., Kieser, T. & Chater, K. F. PCR-targeted *Streptomyces* gene replacement identifies a protein domain needed for biosynthesis of the sesquiterpene soil odor geosmin. *Proc. Natl. Acad. Sci. U.S.A.* **100**, 1541–1546 (2003).
4. Arraj, J. A. & Marinus, M. G. Phenotypic reversal in *dam* mutants of *Escherichia coli* K-12 by a recombinant plasmid containing the *dam*+ gene. *J. Bacteriol.* **153**, 562–565 (1983).
5. Flett, F., Mersinias, V. & Smith, C. P. High efficiency intergeneric conjugal transfer of plasmid DNA from *Escherichia coli* to methyl DNA-restricting *streptomycetes*. *FEMS Microbiol. Lett.* **155**, 223–229 (1997).
6. Gomez-Escribano, J. P. & Bibb, M. J. Engineering *Streptomyces coelicolor* for heterologous expression of secondary metabolite gene clusters. *Microb Biotechnol* **4**, 207–215 (2011).
7. Hopwood, D. A., Kieser, T., Wright, H. M. & Bibb, M. J. Plasmids, recombination and chromosome mapping in *Streptomyces lividans* 66. *J. Gen. Microbiol.* **129**, 2257–2269 (1983).
8. Prentki, P. & Krisch, H. M. *In vitro* insertional mutagenesis with a selectable DNA fragment. *Gene* **29**, 303–313 (1984).
9. Vingadassalon, A. Caractérisation de voies de biosynthèse d’antibiotiques de la famille des pyrrolamides. Université Paris Sud (2013).

10. Gregory, M. A., Till, R. & Smith, M. C. M. Integration site for *Streptomyces* phage phiBT1 and development of site-specific integrating vectors. *J. Bacteriol.* **185**, 5320–5323 (2003).

11. Vingadassalon, A. *et al.* Natural combinatorial biosynthesis involving two clusters for the synthesis of three pyrrolamides in *Streptomyces netropsis*. *ACS Chem. Biol.* **10**, 601–610 (2015).

12. Aubry, C., Pernodet, J.-L. & Lautru, S. A set of modular and integrative vectors for synthetic biology in *Streptomyces*. *Appl. Environ. Microbiol.* AEM.00485-19 (2019). doi:10.1128/AEM.00485-19

13. Kieser, T., Bibb, M. J., Buttner, M. J., Chater, K. F. & Hopwood, D. A. Practical *Streptomyces genetics*. (John Innes Foundation, Norwich, 2000).

14. Jacques, I. B. *et al.* Analysis of 51 cyclodipeptide synthases reveals the basis for substrate specificity. *Nat. Chem. Biol.* **11**, 721–727 (2015).

15. Gil, S., Espinosa, J. F. & Parella, T. IPAP-HSQMBC: measurement of long-range heteronuclear coupling constants from spin-state selective multiplets. *J. Magn. Reson.* **207**, 312–321 (2010).

16. Schmidt, J. M. Asymmetric Karplus curves for the protein side-chain 3J couplings. *J. Biomol. NMR* **37**, 287–301 (2007).

17. Bifulco, G., Dambruoso, P., Gomez-Paloma, L. & Riccio, R. Determination of relative configuration in organic compounds by NMR spectroscopy and computational methods. *Chem. Rev.* **107**, 3744–3779 (2007).

18. Matsumori, N., Kaneno, D., Murata, M., Nakamura, H. & Tachibana, K. Stereochemical Determination of acyclic structures based on carbon-proton spin-coupling constants. A method of configuration analysis for natural products. *J. Org. Chem.* **64**, 866–876 (1999).
